# A multi-layered integrative analysis reveals a cholesterol metabolic program in outer radial glia with implications for human brain evolution

**DOI:** 10.1242/dev.202390

**Published:** 2024-08-27

**Authors:** Juan Moriano, Oliviero Leonardi, Alessandro Vitriolo, Giuseppe Testa, Cedric Boeckx

**Affiliations:** ^1^ Department of General Linguistics, University of Barcelona, 08007 Barcelona, Spain; ^2^ University of Barcelona Institute of Complex Systems, 08007 Barcelona, Spain; ^3^ Human Technopole, Viale Rita Levi-Montalcini 1, 20157 Milan, Italy; ^4^ Department of Oncology and Hemato-Oncology, University of Milan, Via Santa Sofia 9, 20122 Milan, Italy; ^5^ University of Barcelona Institute of Neurosciences, 08007 Barcelona, Spain; ^6^ Catalan Institute for Research and Advanced Studies (ICREA), 08007 Barcelona, Spain

**Keywords:** Indirect neurogenesis, Brain evolution, *Homo sapiens*, KLF6, Cholesterol

## Abstract

The definition of molecular and cellular mechanisms contributing to brain ontogenetic trajectories is essential to investigate the evolution of our species. Yet their functional dissection at an appropriate level of granularity remains challenging. Capitalizing on recent efforts that have extensively profiled neural stem cells from the developing human cortex, we develop an integrative computational framework to perform trajectory inference and gene regulatory network reconstruction, (pseudo)time-informed non-negative matrix factorization for learning the dynamics of gene expression programs, and paleogenomic analysis for a higher-resolution mapping of derived regulatory variants in our species in comparison with our closest relatives. We provide evidence for cell type-specific regulation of gene expression programs during indirect neurogenesis. In particular, our analysis uncovers a key role for a cholesterol program in outer radial glia, regulated by zinc-finger transcription factor KLF6. A cartography of the regulatory landscape impacted by *Homo sapiens*-derived variants reveals signals of selection clustering around regulatory regions associated with *GLI3*, a well-known regulator of radial glial cell cycle, and impacting KLF6 regulation. Our study contributes to the evidence of significant changes in metabolic pathways in recent human brain evolution.

## Teaser

**Highlighted Article:** KLF6 as a regulator of cholesterol in human radial glia with implications for human brain evolution.

## INTRODUCTION

Many studies have unveiled genetic, molecular and cellular features that contribute to species-specific mechanisms of corticogenesis in the primate lineage. These comprise, but are not limited to, transcriptomic divergence, emergence of novel genes, substitutions in regulatory elements, control of the timing of neural proliferation and differentiation, or progenitor diversity and abundance (some recent comprehensive reviews include Pinson and Huttner, 2021; Libé-Philippot and Vanderhaeghen, 2021; Pollen et al., 2023; Vanderhaeghen and Polleux, 2023). In addition, following the availability of genomes from extinct species most closely related to us, the elucidation of the molecular underpinnings of unique aspects of brain organization in *Homo sapiens*, going beyond sheer brain size, is now on the research horizon (Pääbo, 2014), and suggestive evidence for developmental differences is already available (Mora-Bermúdez et al., 2022; Pinson and Huttner, 2021; Stepanova et al., 2021; Trujillo et al., 2021).

The large scale and high resolution afforded by single-cell sequencing technologies, coupled with increasingly powerful computational approaches, have significantly contributed to our understanding of the identity, heterogeneity and developmental progression of neural progenitors. Yet, substantial gaps exist in our knowledge of the regulatory mechanisms implicated in neural progenitor proliferation and differentiation during corticogenesis, and how these mechanisms may have been modified over the course of human evolution.

During neurogenesis, two main proliferative regions can be identified in the dorsal telencephalon. The ventricular zone is populated by ventricular radial glia (vRG), which serve as a scaffold for the growing neocortex as well as a stem cell pool capable of self-renewal and differentiation (Silbereis et al., 2016). And, the subventricular zone (SVZ), which subsequently emerges and expands due to the asymmetric division of vRG and the self-renewal capacity of basal progenitors sustained over a prolonged period (Silbereis et al., 2016). Two main types of basal progenitors can be distinguished: outer radial glial cells (oRG), which retain similar features to vRG, present distinctive morphologies linked to their self-renewal capacity and typically express markers such as *HOPX (*Kalebic and Huttner, 2020*;* Pollen et al., 2015); and intermediate progenitor cells (IPCs), short-lived progenitors with characteristic multipolar morphologies and that express *EOMES* (Pebworth et al., 2021*;* Pollen et al., 2015).

Neurogenesis from basal progenitors, as opposed to the direct route from vRG to neuron, is referred to as indirect neurogenesis, and is thought to be responsible for the generation of the vast majority of upper layer neurons (Lui et al., 2011). Indeed, the developmental period for supragranular layer neuron generation coincides with the appearance of a discontinuous radial glia scaffold where the SVZ remains as the main proliferative niche (Nowakowski et al., 2016). There is growing evidence that the neocortical expansion in the primate lineage that most dramatically affected cortical upper layer neurons, and species-specific features of brain organization, are intimately connected to the regulatory mechanisms that govern the behavior and modes of division of neural progenitor cells (Kriegstein et al., 2006; Rakic, 1995).

Here, we seek to provide a high-resolution characterization of gene regulatory networks (GRNs) at play during indirect neurogenesis and ask whether there is evidence of evolutionary modifications of the (semi)discrete gene expression programs emerging from the modular nature of the regulatory networks we identified. To do so, we leverage an integrative computational framework in which to perform (1) trajectory inference and GRN reconstruction, (2) inference of the dynamics of gene expression programs via the implementation of a new (pseudo)time-informed non-negative matrix factorization method, and (3) a paleogenomic analysis yielding a higher-resolution mapping of the regulatory landscape in which our species acquired derived single nucleotide mutations in comparison with our closest relatives, both extinct and extant, for which high-coverage genomes are available.

Using this framework, we resolve the bifurcation tree defining apical progenitor differentiation towards either oRG or IPCs and characterize waves of gene expression programs activated differentially among the neural lineages leading to each basal progenitor subtype. Among cell type-specific transcription factor (TF)-gene interactions, we uncover a role for TF, *KLF6*, as a putative master regulator of a cholesterol metabolic program specific to the differentiation route leading to oRG. An analysis of TF binding site (TFBS) disruptions leads to the hypothesis of a human-specific regulatory modification of the KLF6-mTOR signaling axis in oRG, with an important role played by TF *GLI3*, for which we identified changes associated with signals of positive selection in our species.

## RESULTS

### Inferring neural progenitor states during indirect neurogenesis from the developing human cortex

**Fig. 1.**
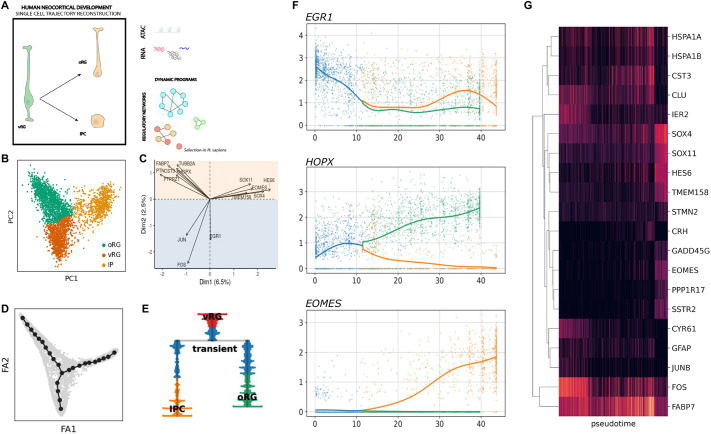
**Resolving the tree of neural progenitor cell differentiation during human corticogenesis.** (A) Schematic of analyses implemented in this paper: single-cell trajectory reconstruction of basal progenitor generation, for the inference and recovery of gene regulatory networks and expression programs, illuminated by paleogenomic analysis. (B,C) Identifying the main axis of variation using principal component analysis (PCA) is a powerful strategy to characterize the heterogeneity and transcriptional dynamics of progenitor cells [as shown for example in a comprehensive study in mice (Mukhtar et al., 2022)]. Here, we performed PCA on a single-cell dataset of human neural progenitors, which allowed the discrimination of radial glia and intermediate progenitor cell subtypes (coarse clustering, B). Top gene loadings with known markers of neural progenitor subtypes are shown in C. (D,E) Inferred tree of principal points and associated dendrogram capturing the hierarchy of neural cell lineage relationships as inferred from single-cell data. (F) Expression trajectory along pseudotime of three marker genes for ventricular radial, outer radial glia and intermediate progenitor cell clusters. (G) Heatmap with representative genes, the trajectories of which significantly change as pseudotime progresses.

Exploiting the potential of high-throughput single-cell sequencing to capture intermediate cellular states during neural cell differentiation, we first sought to characterize the main axis of variation of neural progenitor cells from the developing human cortex at around mid-gestation (Trevino et al., 2021) (Fig. 1A). Principal component analysis (PCA) revealed a marked distinction among cell clusters: the first principal component discriminates among progenitor types, that is, radial glial cells and intermediate progenitors, whereas the second principal component captures the differentiation state, from vRG to basal progenitors (see Fig. 1B). Among genes that contribute the most to each axis, we found markers of progenitor subtypes: e.g., *VIM* and *FOS* for vRG, *HOPX* and *PTPRZ1* for oRG, or *EOMES* and *SSTR2* for IPCs (see Fig. 1C). Coherently, a differential expression analysis on a coarse clustering identified well-known markers for each subtype (Fig. S1). Samples from different batches intermixed in the low dimensional space, confirming the absence of a significant contribution of technical artifacts (Fig. S1A).

To test our ability to reconstruct the apical-to-basal neural lineage trajectories, we performed principal graph learning and computed a force-directed graph where we projected the inferred tree of principal points (Materials and Methods). We obtained a bifurcating tree that resolves the molecular continuum describing the progression of vRG and branching into either oRG or intermediate progenitor fates (Fig. 1D,E). The expression of the aforementioned marker genes recapitulated the expected dynamics along pseudotime (Fig. 1F) as well as that of genes for which the expression trajectories significantly changed along the inferred tree (see Fig. 1G), confirming the differentiation progression through intermediate cellular states. We obtained similar results when an independent dataset was projected into the low dimensional space obtained before via PCA (Polioudakis et al., 2019; Fig. S1C-F). This provides an ideal setting in which to test the validity of our results with time-matched samples around post-conception week 16, a developmental stage with active proliferation in both germinal zones and around the transition from continuous to discontinuous radial glia scaffold (Nowakowski et al., 2016).

### A pseudotime-informed non-negative matrix factorization to identify dynamic gene expression programs

We next sought to characterize how gene expression programs unfold as indirect neurogenesis takes place. A key analytical challenge associated with high-throughput single cell profiling is the ability to extract meaningful patterns from high-dimensional datasets. To overcome this obstacle, we developed a two-step computational strategy aimed at recovering the dynamics of gene expression programs during neural progenitor cell differentiation (Materials and Methods; Fig. S2). Our approach consists of: (1) a pseudotime-informed non-negative matrix factorization (piNMF) as the core algorithm to capture the underlying structure of a high-dimensional dataset, explicitly accounting for the continuous nature of gene expression trajectories through pseudotime, building on recent computational advances on NMF using parametrizable functions (Hautecoeur and Glineur, 2020); and (2) an iterative strategy where stable gene expression programs are recovered by performing K-means clustering over multiple replicates of the matrix factorization core algorithm (following the strategy in Kotliar et al., 2019), thereby addressing the non-uniqueness problem of matrix factorization approximation methods.

**Fig. 2.**
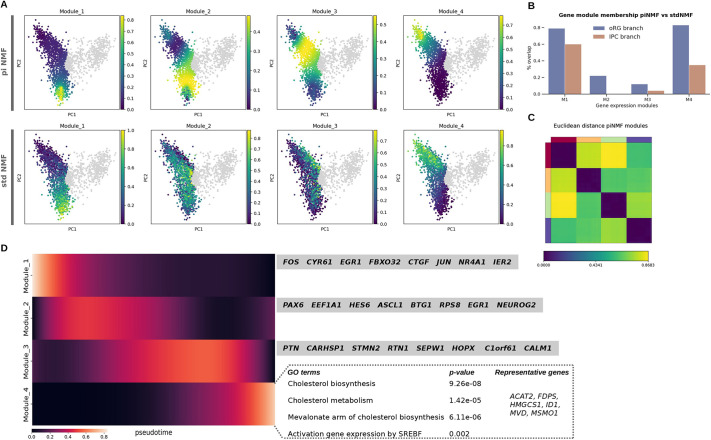
**Pseudotime-informed non-negative matrix factorization recovers a sequential activation of gene expression programs.** (A) Comparatively in PCA plots, piNMF is able to resolve expression programs transiently activated for the lineage branch leading to the outer radial glial cell (oRG) cluster [same for the intermediate progenitor cell (IPC) branch, see Fig. S4], whereas stdNMF does not recover such clear patterns from the data. The scale 0 to 1 denotes activation of each gene expression program in each cell. (B) Genes assigned to modules at the extreme of the lineage tree [ventricular radial glia (vRG) and either oRG or IPC] are shared in higher percentage when compared with modules 2 and 3, confirming that the main differences among non-negative matrix factorization (NMF) algorithms pertain to the transient activation of expression programs along the tree. (C) The high values on the Euclidean distance among the four gene expression programs supports, along with the stability and error measures (see Fig. S4), the factorization rank selection. (D) Heatmap depicting the sequential activation of expression programs in the radial glia branch, with marker genes for each module and, for module 4, representative GO terms highlighted in the main text.

Our strategy departed from the standard NMF (hereafter, stdNMF), where matrix decomposition is achieved through a linear combination of vectors that does not model continuous signals, such as dynamically changing gene expression trajectories. We evaluated the performance of both piNMF and stdNMF approaches on four dominant gene expression programs inferred across cell types and datasets (Materials and Methods; Fig. 2A-C; Fig. S2). Both approaches recovered programs linked to cell cluster identities, which is expected as cell type signatures significantly contribute to the variation detected in single-cell data. However, we observed that expression programs at intermediate states towards basal progenitor clusters were not clearly defined by stdNMF, whereas piNMF finely resolved a sequential activation of expression programs (Fig. 2A). A comparison of statistically significant genes associated to each expression program using multiple least squares regression revealed a higher congruence in gene module membership for programs linked to vRG and oRG cell clusters (especially for oRG, with 79% overlap; 0.35% for IPC) than for transient expression programs (<25%; see Fig. 2B). In line with this, we found that exclusive, top-significant Gene Ontology (GO) terms in transient expression programs captured by piNMF provided a better characterization of key biological processes, with terms that are directly relevant, such as neuroepithelial differentiation, neurogenesis or cerebral cortex, absent in the stdNMF analysis (stdNMF instead returned more generic terms related to cell-cycle and chromatin organization; see Fig. S3).

### A cholesterol metabolic program activated in the radial glial branch

A comparison of expression modules between oRG or IPC clusters inferred via piNMF revealed neural cell biology-specific features. Congruently with the reported roles of gap junctions in coupling radial glial cells (Lo Turco and Kriegstein, 1991), we found GO terms related to cell adhesion and gap junction in the radial glia branch (hypergeometric test; corrected *P*-value<0.05). Similarly, exclusively for the late expression programs (modules 3 and 4) of the radial glia branch, we observed terms related to glia identity such as glia cell projection or glial cell differentiation, as well as terms related to extracellular matrix, crucial for radial glia stemness (corrected *P*-value<0.05; Fietz et al., 2012; Pollen et al., 2015). Among the exclusive terms overrepresented in the IPC branch we found G1 phase, including a key regulator of basal progenitor G1 phase-length cyclin D1 (Lange et al., 2009), cell-cell signaling and Notch signaling (Kawaguchi et al., 2008), as well as axon and cell projection terms [in agreement with a reported activation of axogenesis-related genes in basal progenitors in mouse (Bedogni and Hevner, 2021); all significant *P*-values can be found in Table S1]. These results indicate that the piNMF implemented here successfully captured relevant molecular processes during neural cell differentiation.

Prominently, the module that is activated last in pseudotime and that pertains to the acquisition of oRG identity returned an overrepresentation of genes involved in cholesterol metabolism (corrected *P*-value<0.01; Fig. 2D). For example, we observed the activation of the expression of several enzymes of the cholesterol biosynthesis pathway, such as the 3-hydroxy-3-methylglutaryl-coenzyme A (HMG-CoA) synthase 1, which participates in a condensation reaction before production of the cholesterol precursor mevalonate, or the mevalonate pyrophosphate decarboxylase (MVD), which catalyzes the production of isoprenes for cholesterol synthesis. Although the interplay of cholesterol metabolism and neural progenitor cells still awaits systematic exploration (Namba et al., 2021), previous studies using mice have revealed important roles for cholesterol in the context of cortical radial thickness and neural stem cell proliferation and differentiation (Corbeil et al., 2010; Nourse et al., 2022; Saito et al., 2009).

The prominence of cholesterol metabolism in the oRG cluster, absent from IPC cluster gene expression modules, was replicated when analyzing an independent dataset (Polioudakis et al., 2019) and additionally cross-validated by GO terms that were also captured by the standard NMF despite gene module composition differences (Tables S1 and S2). To further strengthen our results, we integrated our reference dataset with an openly available atlas of neocortical development (Bhaduri et al., 2021) that allowed us to widen our analysis on spatiotemporally matched prefrontal cortical samples to also encompass the visual cortex (Fig. S5A). We recapitulated the apical-to-basal progenitor bifurcation trajectory (Fig. S5B,C) and, in line with the above, significant GO categories related to acquisition of oRG fate in the integrated dataset were related to lipid, fatty acid transporters and membrane organization (corrected *P*-value<0.05; Table S2).

### A KLF6-centered regulatory network for the activation of a cholesterol metabolism program in human radial glia

**Fig. 3.**
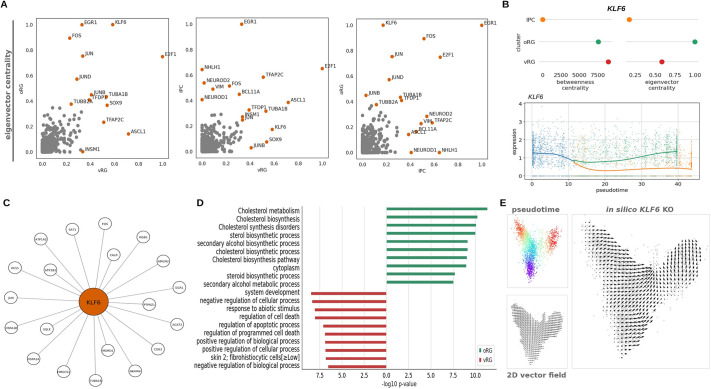
**Gene regulatory network reconstruction from human neural progenitor single-cell data.** (A) Pairwise comparisons of eigenvector centrality values among single-cell progenitor cell clusters, highlighting top 10 genes in each cluster. Some differentially expressed genes for the ventricular radial glia (vRG) cell cluster retain some level of expression in basal progenitors and are indeed present among top ten genes for different gene regulatory network (GRN) connectivity measures across clusters (see also Fig. S7); this is the case for *EGR1*, *FOS* or *JUN*. In addition to *KLF6* for outer radial glial cells (oRG), other genes that are more prominently associated with specific clusters include *ASCL1*, *SOX9*, *TFAP2C* for vRG when compared with oRG, or neuron differentiation-related basic helix-loop-helix transcription factors *NEUROD1*, *NEUROD2* and *NHLH1* between intermediate progenitor cell (IPC) and radial glia clusters, consistent with the closer transcriptomic similarity of IPCs to excitatory neurons (Bhaduri et al., 2021). (B) *KLF6* network measures across single-cell clusters, with a marked contrast between IPC and RG clusters, and most prominently as central node in oRG (eigenvector centrality). Below, *KLF6* expression along pseudotime, showing upregulation in oRG and downregulation in IPC. (C) Top representative genes by network weight among KLF6 target genes. (D) Top GO terms associated with KLF6 targets in oRG and vRG, with prominence of cholesterol metabolism in oRG. Cholesterol metabolism GO terms only appear for vRG cluster KLF6 targets if relaxing the *P*-value threshold above 0.01 (see also Table S3). (E) A vector field represents the predicted bifurcation trajectory from apical (low pseudotime values) to basal (high pseudotime values) progenitors. The *in silico* perturbation of *KLF6* predicts a depletion of both oRG and vRG, with a cell fate shift towards IPC.

We next proceeded to the identification of key regulators of gene expression programs active during neural progenitor cell fate dynamics. We performed a GRN reconstruction using the CellOracle software (Kamimoto et al., 2023). First, we identified replicated signals across single-cell ATAC-seq studies on the developing human brain in order to create a brain atlas of open chromatin regions (Materials and Methods). Second, we retained confident TF-target gene links from the open chromatin region atlas for each cell cluster, based on a machine learning-based regression analysis on the single-cell gene expression data (Materials and Methods). We evaluated the prominence of TFs and genes within the reconstructed networks for each progenitor subtype cluster according to the following network connectivity measures [as proposed in Kamimoto et al. (2023)]: eigenvector centrality, for overall relevance of a given gene in a network according to the quality of its connections to other genes, and betweenness centrality, i.e. the influence of a given gene in the transfer of information within a network. Consistently across network measures and comparatively among cell clusters, we found the zinc finger-containing TF *KLF6* as one of the top-ranked genes in radial glial cells (Fig. 3A,B; Fig. S7A). This is consistent with the association of the gene to a super-interactive promoter in radial glia (Song et al., 2020), but not in IPCs. Within radial glia, KLF6 occupies a more prominent position in the oRG cluster [these results were replicated in an independent dataset (Polioudakis et al., 2019); Fig. S7A,B].

To gain further insight into the cell cluster-specific regulatory network associated with KLF6, we compared its target genes in vRG and oRG cell clusters. KLF6 targets in vRG are most significantly related to biological processes that include responsiveness to abiotic stimulus and organic substances, regulation of apoptosis, neurogenesis or cell migration (corrected *P*-value<0.01). By contrast, in the oRG cluster, the KLF6 transcriptional network is significantly over-represented in genes linked to cholesterol and steroid biosynthesis, as indicated by GO terms such as cholesterol metabolism, regulation of cholesterol biosynthesis by SREBF and steroid biosynthesis or steroid metabolic process (corrected *P*-value<0.01; Fig. 3C,D; Table S3). To test for temporal differences in gene expression, we compared early and late radial glia at neurogenic stages and did not identify *KLF6* or *KLF6* cholesterol-related genes as statistically significant differentially expressed genes (Fig. S4D).

We performed a similar analysis on an independent dataset (Polioudakis et al., 2019) and, although we did not obtain a clear discrimination for KLF6 roles in radial glia cell subtypes (with few terms related to steroids in radial glia; Table S3), when we examined the KLF6 transcriptional network reported in Polioudakis et al. (2019), an enrichment for cholesterol metabolism emerged (corrected *P*-value<0.01; Table S3). Additionally, we observed a recapitulation of the statistically significant GO terms related to oRG fate (cholesterol biosynthesis pathway, or cholesterol metabolism; corrected *P*-value<0.05) when analyzing the integrated dataset [reference dataset with prefrontal and visual cortical samples from Bhaduri et al. (2021)]. However, in this case, we found a more similar profile in vRG as well (Table S3).

We found KLF6 target genes across the four sequentially activated gene expression programs detected by piNMF, and specifically enzymes of the cholesterol biosynthetic pathway in the latest-activated module in oRG (corrected *P*-value<0.01). As expected, KLF6 targets present in piNMF modules were enriched in cholesterol metabolism exclusively in the latest oRG module (Table S4). Lastly, in agreement with the reported roles of KLF6 as a regulator of cholesterol metabolism via activation of mTOR signaling and sterol regulatory element binding TFs (Syafruddin et al., 2019), we detected the mTOR signaling-related platelet-derived growth factor receptor PDGFRB and insulin-like-growth factor binding protein IGFBP2 as well as the GO term ‘activation of gene expression by SREBF′ in the late piNMF module 4 (corrected *P*-value<0.01; Table S4; see also Table S5).

To further test the central roles of KLF6 regulatory programs on radial glia, we leveraged the GRN modeling from CellOracle framework to perturb *KLF6* expression *in silico* (Fig. 3E). This *KLF6* knockout simulation reveals a prominent impact on radial glia fate, with a shifting in cell state trajectories towards IPs. Taken together, our results reveal a TF, KLF6, acting as a central node for the activation of a cholesterol metabolic program in human radial glia.

### A paleogenomic interrogation of regulatory regions active during human corticogenesis

In light of recent work mentioned in the introduction showing how some protein-coding mutations (virtually) fixed across contemporary human populations but absent in closely related extinct species affect various aspects of neural progenitor cell behavior, and especially metabolic programs, we decided to take advantage of our comprehensive atlas of open chromatin regions active during human corticogenesis presented above and an extensive catalog of derived changes in our lineage (Kuhlwilm and Boeckx, 2019) to focus on the still less well studied mutations in the regulatory regions of the genome, aiming to identify any points of divergence among closely related species that achieved similar brain sizes (VanSickle et al., 2020), but likely via distinct ontogenies (Hublin et al., 2015), reflected in different neurocranial shapes.

**Fig. 4.**
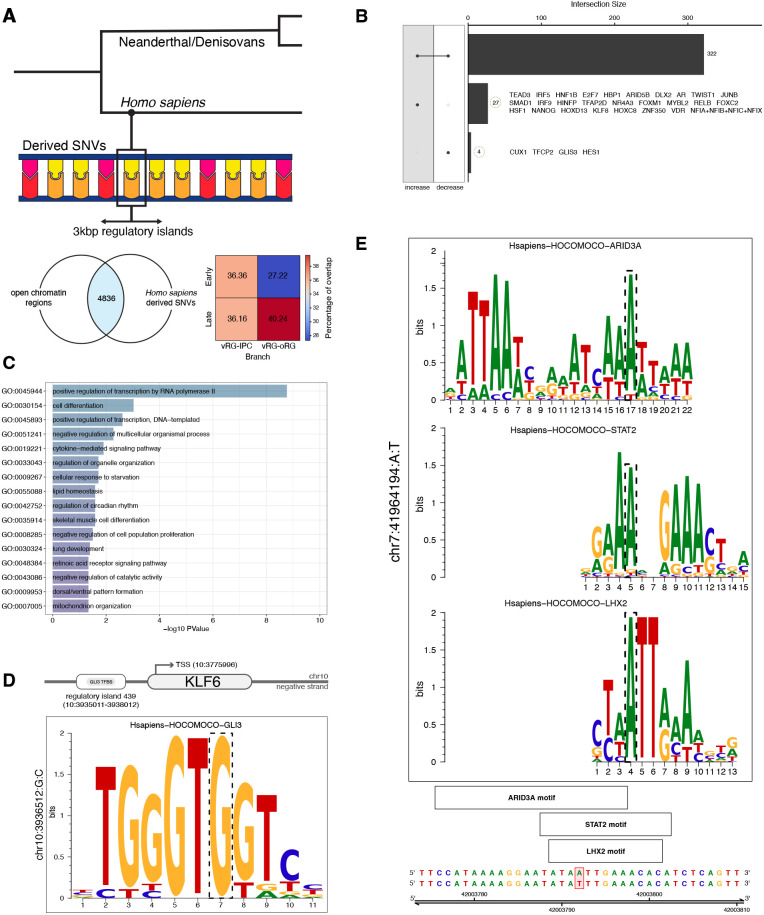
**Paleogenomic analysis of regulatory variants.** (A) Building on the brain atlas of open chromatin regions, regulatory islands were defined as 3 kbp-length regions where *Homo sapiens* acquired derived alleles in comparison to Neanderthals and Denisovans (carrying the ancestral version found in chimpanzees). Genes associated with regulatory islands are found in pseudotime-informed non-negative matrix factorization (piNMF) modules detected for both ventricular radial glia (vRG) to intermediate progenitor cell (IPC) and vRG to outer radial glia (oRG) branches, with more pronounced abundance on the oRG lineage. (B) Overview of the changes in transcription factor (TF) binding affinity scores to target sites harboring *Homo sapiens*-derived regulatory variants. (C) GO enrichment analysis for TFs whose binding affinity is impacted by *Homo sapiens*-derived regulatory variants. (D) Schematic visualization of the predicted TF differential regulation of *KLF6* by GLI3, with TF motif impacted by a *Homo sapiens*-derived single nucleotide variant (dashed box). (E) Schematic visualization of TF binding sites with *Homo sapiens*-derived mutations highlighted in the context of *GLI3* (dashed box).

To do so, we first isolated a set of regulatory regions that contain high-frequency *Homo sapiens*-derived variants but, crucially, for which the Neanderthals/Denisovans carry the ancestral allele [following the criteria in Kuhlwilm and Boeckx (2019)]. We call these ‘regulatory islands’, and defined such regions in terms of a genomic window of 3000 base pairs around each variant where the Neanderthal/Denisovan homolog regions did not acquire species-specific, derived variants (Fig. 4A; Materials and Methods). This led to the identification of a total of 4836 regulatory islands linked to 4797 genes, complementing and extending recent efforts on regulatory variants derived in our lineage (McArthur et al., 2022 preprint; Moriano and Boeckx, 2020; Weiss et al., 2021).

Next, we tested whether regulatory regions associated with genes found in either oRG- or IPC-lineage gene expression modules identified in our above analysis were statistically associated to regulatory islands more often than by chance (Materials and Methods). An enrichment for regulatory regions of genes in the IPC lineage in regulatory islands was detected (permutation test, *P*-value<0.01), although this was not the case for the oRG lineage (*P*-value=0.24). We caution, however, that the genes tested (highly variable genes) are only a subset of those expressed by neural progenitor cells. In terms of relative proportions, we observed a more pronounced abundance of genes associated with regulatory islands in late, relative to early, modules in the oRG branch, whereas a more even distribution is observed in the IPC branch [Fig. 4A; for both datasets studied here, Trevino et al. (2021) and Polioudakis et al. (2019); see also Table S6]. Among the genes linked to regulatory islands we found key oRG markers such as *HOPX*, *PTPRZ1*, *LIFR*, *MOXD1*, and indeed *KLF6*, to which we return in the next subsection.

We then evaluated whether regulatory islands are enriched in two genomic regions of special relevance for the recent evolution of *Homo sapiens*: genomic regions depleted of archaic introgression (so-called large ‘introgression deserts’) (Chen et al., 2020) and regions under putative positive selection (Peyrégne et al., 2017). We detected a significant enrichment for regulatory islands within positively-selected regions (permutation test, *P*-value<0.01), in line with previous results indicating that putative positively-selected regions in our genome are enriched in regulatory regions (enhancers) (Peyrégne et al., 2017). No such result was found for regulatory islands and deserts of introgression (under-representation with *P*-value<0.01). These intersections bring to relevance predicted direct targets of KLF6 present in deserts of introgression, *PTPRZ1* and *RB1CC1*, as well as interacting regulators for cholesterol biosynthesis (Sun et al., 2005; Yang et al., 2002), such as *SCAP* [with a fixed derived missense mutation in *Homo sapiens*; Kuhlwilm and Boeckx, (2019)] and *SEC24D*, exhibiting signals of positive selection in our species (Table S6).

### Differential transcription factor binding analysis

Differential TF binding plays a key role in the divergence of gene regulation across species (Villar et al., 2014; Zhang et al., 2023), and indeed *Homo* species-specific regulatory variants have been associated with differential gene expression in cell-line models (Weiss et al., 2021). We performed a systematic evaluation of TF motifs that are found in regulatory islands by implementing the motifbreakR predictive tool (Coetzee et al., 2015). Specifically, we tested whether variants at TFBS are responsible for TF differential binding affinity, and asked whether an overall reduced, increased or unchanged binding affinity is detected.

After filtering the results based on the distribution of affinity difference scores, out of 400 TFs in the Hocomoco collection, we found 27 with overall increased affinity and four with reduced affinity, and 322 TFs showed both an increase and a decrease in binding affinity at different sites (Fig. 4B). To functionally interpret the biological roles of TFs whose binding sites are impacted by *Homo sapiens*-derived mutations, we performed a GO term enrichment analysis and observed, among the top significant biological processes (*P*<0.05), lipid metabolism and also inflammation-related signaling pathways (Fig. 4C; Table S7).

Interestingly, a rank of TFs with highest number of increased binding affinity sites revealed statistically significant GO terms related to the regulation of the adaptive response to hypoxia and various metabolic processes including lipid metabolism (*HIF1A*, *ARNT*), and included a prominent downstream target of KLF6 in the regulation of cholesterol metabolism, *BHLHE40* (Syafruddin et al., 2019), prominently so in regulatory islands associated with signals of positive selection. Here, it is noteworthy that regulatory islands affected by differential BHLHE40 binding include target genes such as *GLI3* as well as *ITGB8*, implicated in PI3K-AKT-mTOR signaling in (outer) radial glia (Mora-Bermúdez et al., 2016; Pollen et al., 2019). Another TF controlling cholesterol homeostasis, SREBF2, exhibits differential binding affinity for a regulatory island linked to PALMD, which plays a specific role in basal progenitor proliferation (Kalebic et al., 2019).

Next, we decided to focus on differential binding affinity sites impacting *KLF6*, given its prominence in our previous results. Our analysis predicts a *KLF6*-associated regulatory variant altering a GLI3 TFBS (chr10:3936512-G-C, hg38 genome version), with higher affinity in *Homo sapiens* when compared with the ancestral variant found in Neanderthal/Denisovan genomes (Fig. 4D; Table S8). Given the mutual regulation of cholesterol and sonic hedgehog signaling (Blassberg and Jacob, 2017; Wang et al., 2016), we found this differential binding affinity by GLI3 particularly intriguing: *GLI3* is a crucial regulator of the dorsoventral cell fate specification and the switch between proliferative and differentiative radial glia divisions [in different model systems (Fleck et al., 2023; Hasenpusch-Theil et al., 2018)].

We found two regulatory islands under positive selection linked to *GLI3*, which is one of the genes for which the expression trajectory significantly changes through pseudotime. In fact, our piNMF analysis placed *GLI3* prominently at the juncture between early and late radial glia modules (program 2; indeed, the beginning of the late oRG piNMF modules includes GO term ‘hedgehog offstate’; Table S4). In addition, regulatory islands linked to *GLI3* and associated with positive selection already mentioned above are associated with increased binding affinity for mTOR signaling related genes *STAT2*, a cytokine regulator implicated in cell proliferation control and inflammation response (Ho et al., 2016), *ARID3A* and *LHX2*, both modulators of the cell cycle and the tempo of cortical neurogenesis (Hsu et al., 2015; Saadat, 2013; Suresh et al., 2024) (Fig. 4E; Table S8).

Finally, it is noteworthy that the GLI3 variants within regulatory islands under putative positive selection have ClinVar-associated phenotypes (Landrum et al., 2018), with the minor (ancestral) allele linked to Greig cephalopolysyndactyly syndrome (OMIM: 175700) and Pallister Hall syndrome (OMIM: 146510), which affect brain size and craniofacial traits among other clinical features. Validating the impact of these changes in an experimental setting is an important research direction emerging from this analysis. We observe in this context that within the KLF6 transcriptional networks in our analysis one finds prominent GLI3 targets relevant for the specification of dorsal telencephalic progenitors (Fleck et al., 2023), such as *HES1*, *HES4* or *HES5*, as well as *CTNNB1*. In addition, experimental perturbation of GSK3β, a kinase that integrates multiple signaling pathways (including hedgehog and WNT-β-catenin signaling in mice neural progenitors; Kim et al., 2009), specifically affects cholesterol metabolism and indeed *KLF6* expression coincident with the emergence of the oRG lineage in human cortical organoids (López-Tobón et al., 2019).

## DISCUSSION

Previous large-scale single-cell studies have extensively characterized neural cells from the developing human brain. However, the molecular definition of the lineage tree relating apical progenitors to basal progenitor populations, as part of an intricate web of complex lineage relationships, has remained elusive. By implementing an integrative computational framework for the joint investigation of different biological layers of the cell using high-throughput single-cell data, we characterized gene expression programs sequentially activated during progenitor cell progression and identified key transcriptional regulators, shedding light onto central processes of neural progenitor cell fate dynamics and evolutionary modifications thereof.

Our findings uncover KLF6 as a central node in human radial glia transcriptional networks. KLF6 is a member of the zinc finger-containing family of TFs resembling *Drosophila* protein Krüppel (Dang et al., 2000), but its role in human neurogenesis has to date remained largely undescribed. *KLF6* has been associated with a ‘super-interactive’ promoter specifically in radial glia (Song et al., 2020) and its targets during neocortical development have been reported to be enriched in oRG (Polioudakis et al., 2019), consistent with our findings based on GRN reconstruction and piNMF. We identified several enzymes implicated in cholesterol biosynthesis under the KLF6 transcriptional control, prominently during the acquisition of oRG identity. However, given the expression and centrality of *KLF6* in vRG, the cell type-specific functional roles of *KLF6* requires further investigation. Previous studies in other model systems have also reported similar gene expression programs regulated by KLF6 related to lipid homeostasis (Syafruddin et al., 2019; Wang et al., 2018).

Future work is required to elucidate the roles of cholesterol metabolism in oRG proliferation and neurogenesis, particularly in light of clinical association of *KLF6* to glioblastoma (Masilamani et al., 2017), in which sustained cholesterol synthesis impacts tumor cell growth (Kambach et al., 2017). We suspect it will be particularly productive to examine the role of cholesterol biosynthesis in the context of immune/inflammation regulation. oRG are known to have specific energetic demands related to aerobic glycolysis that are reminiscent of inflammation phenotypes (Soto–Heredero et al., 2020) (also associated with hyperactivation of the mTOR pathway; Allan, 2008). Several key oRG markers, such as STAT3, IL6ST and *LIFR* (Pollen et al., 2015) have a well-established role in immunity/inflammation control. Interestingly, several of the genes related to cholesterol biosynthesis highlighted in our analysis, such as *SREBF2*, *BHLHE40* and indeed *KLF6* have been shown to be involved in immune modulation by cholesterol and its regulation of the endothelial response to cytokines (Fowler et al., 2023) (a significant GO term in our analysis of TF binding site modifications; Fig. 4).

The metabolic control of neural progenitor cell behavior significantly contributes to species-specific features of brain evolution (Iwata and Vanderhaeghen, 2021; Namba et al., 2021), and experimental evidence already points to significant changes impacting various metabolic pathways in our recent evolution (after the split from our closest extinct relatives) (Pinson et al., 2022; Stepanova et al., 2021). Our evolutionary-informed analysis of TFBS disruptions contributes to this emerging picture by highlighting modifications clustering around cholesterol metabolism. In addition, our study highlights the relevance of mutations affecting *GLI3*. Not only did we infer a differential regulation of *KLF6* by GLI3, we also uncovered regulatory islands associated with signals of positive selection predicted to impact *GLI3* expression during cortical development. Previously, a study on cortical organoids identified a human differentially accessible region linked to *GLI3* when compared with chimpanzee organoids (Kanton et al., 2019).

We find it noteworthy that some of the variants defining the regulatory island around *GLI3* are among the most recent derived high-frequency *GLI3* changes in our lineage (Kuhlwilm and Boeckx, 2019), and are predicted to have emerged between 200 and 300 kilo years ago (kya; Andirkó et al., 2021 preprint), a significant period in our recent evolutionary history (Hublin et al., 2017; Schlebusch et al., 2017; Skoglund et al., 2017). Also, in light of our findings, future research may explore further the promising interplay between the primary cilia and GLI3 activity in the regulation of cell cycle length and cortical size (Wilson et al., 2012), considering as well the evolutionarily relevant role of mTOR signaling in ciliary dynamics, impacting basal progenitors in particular (Heurck et al., 2023), and between cholesterol accessibility and the regulation of hedgehog signaling in the membrane of the primary cilium (Kinnebrew et al., 2019).

Our approach illustrates the relevance of paleogenomes in adding temporal precision to important differences that comparisons between humans and other great apes already revealed (Pollen et al., 2023), in particular here the role of mTOR signaling in human cortical development (Pollen et al., 2019). At a more general level, our work adds to the mounting evidence for the importance of regulatory regions in modifying developmental programs in the course of (recent) human evolution (Gokhman et al., 2020; Kaplow et al., 2023; Keough et al., 2023; Mangan et al., 2022; Moriano and Boeckx, 2020; Peyrégne et al., 2017; Weiss et al., 2021).

Our work also shows how paleogenomics offers the potential to probe questions about brain evolution that go beyond traits that may be recoverable from the (traditional) fossil record, such as overall adult brain size or shape. Our evolution-oriented analysis invites the hypothesis that important modifications impacting upper-layers of the neocortex took place relatively recently in our history. The evidence presented here involving differential regulation of cholesterol signaling in oRG, together with independent evidence concerning changes affecting genes specifically involved in basal progenitor proliferation [such as *PALMD* (Kalebic et al., 2019; Kuhlwilm and Boeckx, 2019) or *TKTL1* (Pinson et al., 2022)], as well as upper-layer neuron markers like *SATB2* (Weiss et al., 2021), points to the need to probe the nature of associative, cortico-cortical connections characteristic of upper-layer neuronal ensembles further.

## MATERIALS AND METHODS

### Single-cell RNA-seq data processing

Raw single-cell RNA-seq datasets from selected studies were processed using Seurat 4.2.0, guided by best practices of single cell analysis (Butler et al., 2018; Luecken and Theis, 2019; Stuart et al., 2019). Seurat objects were created from raw count matrices and retention of high quality cells was based on the following cell attributes: total counts, expressed genes, percentage of mitochondrial gene counts and percentage of zero counts, requiring a distribution of values within three median absolute deviations for each attribute and per batch. Actively dividing cells were filtered out based on *TOP2A* expression. To jointly analyze samples from different batches, as well as data from Trevino et al. (2021), Polioudakis et al. (2019) and Bhaduri et al. (2021), in a shared low dimensional space, we performed data normalization with Seurat dedicated function SCTransform, and then followed strategy presented in Stuart et al. (2019) to identify a set of anchor cells (‘FindIntegrationAnchors’ function) for the integration of datasets (‘IntegrateData’ function), before computing PCA. A common processing was implemented for inferring the branch trajectories and for GRN reconstruction (see below): retaining genes with expression in at least 50 cells, normalization of cell counts to equal median of counts per cell before normalization, selection of 4000 highly variable genes based on Seurat variance-stabilizing transformation algorithm (Hafemeister and Satija, 2019), followed by re-normalization and log-transformation. Coarse clustering was performed using Leiden algorithm and resolution parameter to 0.1. Logistic regression was used to identify differentially expressed genes. Cell cluster annotation was based on both differential expression analysis and available annotations from the original publications. A synthetic dataset was generated using dynverse trajectory inference tool (Cannoodt et al., 2021) for a bifurcating model with number of cells set to 1000, 2000 features and dropout probability factor 1000. Differential expression analysis was performed using a Wilcoxon rank rum test as implemented in Seurat, setting a log fold-change threshold of 1.25, genes detected in at least 0.25% of cells in each cluster, and a minimum gene detection difference between clusters of 0.5; differences were considered significant if adjusted *P*-value<0.01.

Complementarily, we performed single-cell trajectory reconstruction using python package scFates (Faure et al., 2023) on normalized, log transformed count matrices. A force-directed graph was drawn using our previously computed PCA coordinates for initialization. Then we used the Palantir software (Setty et al., 2019) included in the scFates toolkit to generate a diffusion space for tree learning using the ElPiGraph algorithm. Pseudotime was calculated using *FOS* gene expression for root selection and the genes that significantly changed in expression along the inferred tree were identified using the scFates cubic spline regression function.

### Gene regulatory network inference and analysis

GRN reconstruction was performed following the computational framework of CellOracle software (Kamimoto et al., 2023), combining single-cell ATAC-seq and RNA-seq data modalities for TF-target genes inference.

In order to build an atlas of open chromatin regions active during human cortical development, we selected as reference the singleome ATAC-seq dataset from Trevino et al. (2021), containing the highest number of ATAC-seq peaks, and required a minimum of 50% overlap with open chromatin signals from one of the following datasets: multiome ATAC-seq data from Trevino et al. (2021) or ATAC-seq datasets from Markenscoff-Papadimitriou et al. (2020) and de la Torre-Ubieta et al. (2018). As the reference dataset does not contain signals for the X and Y chromosomes, we included these data as available in Markenscoff-Papadimitriou et al. (2020) and de la Torre-Ubieta et al. (2018). A total of 392,961 regulatory regions (hg38 genome version) were used for downstream analyses. We then built regulatory region-gene associations based on genomic proximity and literature curated regulatory domains (McLean et al., 2010). Next, we scanned each regulatory region for TF motifs using the Hocomoco database version 11 (Kulakovskiy et al., 2018). The resulting TF-regulatory region-gene associations represent the raw GRN for the machine learning-based regression analysis to impute cluster-specific GRNs (Kamimoto et al., 2023). Of the two algorithms available in the CellOracle software, we chose the bagging ridge regression model, as it consistently reported better scores for network degree distribution (Fig. S6). Cluster-specific TF-target gene interactions were obtained by filtering by a *P*-value threshold of 0.001 for connection strength and a maximum of 2000 links per cluster. An evaluation of such GRNs was performed on the basis of the centrality measures, including betweenness centrality and eigenvector centrality, as proposed in Kamimoto et al. (2023). GO enrichment analysis, as for evaluating NMF results (see below), was performed using python package of g:Profiler (Kolberg et al., 2023). Results were considered significant if hypergeometric tests reported corrected *P*-value<0.05.

### Pseudotime-informed non-negative matrix factorization

Matrix factorization techniques aim to infer the underlying structure of a high dimensional dataset and to provide interpretable meaningful components, thus with diverse applications on high throughput data, including the inference of gene expression programs (Stein-O'Brien et al., 2018). Specifically, we implemented a matrix factorization analysis to learn the dynamics of gene expression programs dependent on pseudotime from single-cell data. We applied a non-negative matrix factorization that comprises the decomposition of a matrix of *n* vectors with non-negative values into two lower rank, non-negative matrices: the pattern matrix containing basis vectors and the coefficient matrix with the coefficients of the non-negative linear combination of the basis vectors, aiming to minimize:
(1)

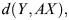
where *d* is the distance (by a given measure) between the original matrix and the reconstruction AX. As our inquiry deals with cellular differentiation events, we sought to decompose a high dimensional single cell dataset accounting for the dynamic nature of gene expression trajectories through pseudotime. As the core algorithm, we computed the matrix factorization following the original work of Hautecoeur and Glineur (2020), where the approximation is now:
(2)

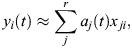
where each vector of *y* is a function dependent on time *t*, *a* contains a set of *r* non-negative functions, and *x* contains the non-negative coefficient values, for a given factorization rank *r* and 1≤*j*≤r*,* 1≤*i*≤*n*. As with other factorization methods, there is no *a priori* knowledge of the factorization rank (i.e. expected number of patterns in the data), and thus *r* must be provided by the user; measures of stability and error (see below) can guide this selection. Here, we chose four expression programs as a neat balance between stability across branches and datasets and resolution of semi-discrete modules along pseudotime (see Fig. 2 and Fig. S4). We used degree 3 splines as the set of functions to model gene expression trajectories, selecting the number of knots (obtaining intervals where to fit trajectories) to 4 (a low number avoids overfitting and better captures global trends). The algorithm to solve the factorization problem is based on Hierarchical Alternating Least Squares [implemented in Hautecoeur and Glineur (2020)], and a maximum number of iterations of 10^4^ and tolerance 10^−10^ were set as stopping criteria.

Given that NMF is a matrix approximation method, we followed the iterative and clustering strategies presented in Kotliar et al. (2019) as an extended algorithm to recover stable gene expression modules. Matrix decompositions from the core algorithm presented above were computed over 750 iterations per factorization rank to obtain replicates that were then clustered via KMeans clustering based on Euclidean distance to obtain consensus values for the pattern and coefficient matrices. Measures of stability and error of the matrix reconstruction were calculated using silhouette scores and the Frobenius norm of approximation, respectively, following Kotliar et al. (2019). Additionally, in order to statistically associate genes to gene expression programs, marker genes for each module were identified using the normalized *z*-score gene expression value of each gene for multiple least squares regression against the programs in the pattern matrix, as implemented in Kotliar et al. (2019). We refer to (semi)discrete modules to indicate that genes might be present in more than one module to the extent the association is statistically significant (higher expression than the rest of genes in cells with activation of the given expression module).

### Paleogenomic analysis

We made use of a paleogenomic dataset (Kuhlwilm and Boeckx, 2019) that catalogs segregating sites between *Homo sapiens* and high quality genomes from two Neanderthals and one Denisovan individual (Meyer et al., 2012; Prüfer et al., 2017; 2014), where ancestrality was inferred from publicly available multiple genome alignments (Paten et al., 2008) or, when this information was not available, from the macaque reference genome (Yan et al., 2011). Allele frequency was determined from the dbSNP database build 147 (Sherry et al., 2001) and a 90% allele frequency threshold was set to retain high-frequency variants for further analyses. In the search for regulatory regions that might have been under selection in recent *Homo sapiens* evolution and that differentially impact gene expression, we intersected the regulatory regions from our open chromatin region brain atlas with *Homo sapiens*-derived variants where the Neanderthals/Denisovans carry the ancestral allele (using the bedtools suite; Quinlan and Hall, 2010). Additionally, to identify genomic regions that may encapsulate *Homo*-specific regulatory mechanisms, we required for each variant to be contained within a genomic window of at least 3000 bp where the Neanderthal/Denisovan homolog regions did not accumulated lineage-specific derived changes. A total of *n*=4836 regulatory islands were identified and associated to 4797 genes. To detect signals of selection, we intersected genome coordinates of regulatory islands with putative positively-selected regions identified by Peyrégne et al. (2017) as unusually long genomic regions that contain variants that reach high or even fixation in our species after our divergence from the Neanderthal/Denisovan lineage. A similar approach was used to identify regulatory islands within regions that are significantly depleted of Neanderthal/Denisovan ancestry (Chen et al., 2020). Permutation tests were performed using R package regioneR (Gel et al., 2016), setting number of iterations to 10,000 and using random genomic regions of similar size as control for each test.

To evaluate disruptions of TFBS, we generated a set of genomic coordinates of variants sitting within regulatory islands using a unique identifier based on genomic coordinates and allele information. Differences in TF binding affinity were computed, applying the information content method from the motifbreakR package (Coetzee et al., 2015) and using position weighted matrices annotated in the Hocomoco motif collection (Kulakovskiy et al., 2018) (consistent with our GRN reconstruction analysis). A significance threshold was set to 1e-4 and an even background nucleotide distribution was assumed. The *P*-values were then adjusted for multiple testing using the Benjamini-Hochberg method. Redundant motifs were dropped and the resulting TF-variant associations further filtered by retaining only those with a predicted affinity difference falling in the fourth quantile of the distribution. Finally, a frequency score was computed for each TF based on the number of strong over total hits identified. GO enrichment analyses were performed on the TF identified as described above (using the same Hocomoco motif collection as custom reference set). Analyses were performed with the TopGO package (Alexa et al., 2006) using the following parameters: ‘weight01’ as algorithm, ‘Fisher’ as statistics, 8 as ‘nodeSize’ and 3 as ‘minTerms’; a *P*-value<0.05 and an enrichment>1 were set as thresholds to select significant GO terms.

### Limitations

The (pseudo)temporal ordering of gene expression states from single-cell data presented here allows us to interpret cell differentiation as a molecular continuum, but it remains to be seen how closely this recapitulates the transcriptional dynamics of lineage progression *in vivo*. Additionally, the process of indirect neurogenesis studied here idealizes away from what is a much more complex network of lineage relationships among neural progenitor subtypes. The reconstruction and recovery of regulatory networks and expression programs rely on the identification of a set of TFs and highly variable genes that only partially represent the higher complexity of the cells. This complexity is even more manifest when the temporal differences among neural progenitors during the long human gestational period is taken into account. Lastly, future experimental work is required to validate the predictions derived from the paleogenomic interrogation of regulatory variants presented here.

## Supplementary Material



10.1242/develop.202390_sup1 Supplementary information

Table S1. Gene ontology enrichment results for NMF gene expression modules - reference dataset.

Table S2. Gene ontology enrichment results for NMF gene expression modules - testing dataset and integration.

Table S3. Gene ontology enrichment results for cell type-specific KLF6 regulatory networks.

Table S4. Gene ontology enrichment results for KLF6 targets across piNMF gene expression modules.

Table S5. Comparison gene regulatory networks across datasets and cell types.

Table S6. Associated genes to regulatory islands, deserts of introgression, and positively selected regions across early and late gene expression modules.

Table S7. Gene ontology enrichment results for TFs impacted by *Homo sapiens*-derived variants.

Table S8. TF differential binding affinity analysis results.
