# Supplementary material for: A multi-layered integrative analysis reveals a cholesterol metabolic program in outer radial glia with implications for human brain evolution
Source: Development. 2024 Aug 27;151(16):dev202390. doi: 10.1242/dev.202390 (PMC11385646; doi:10.1242/dev.202390)
Supplement: Supplementary information [file develop-151-202390-s1.pdf]

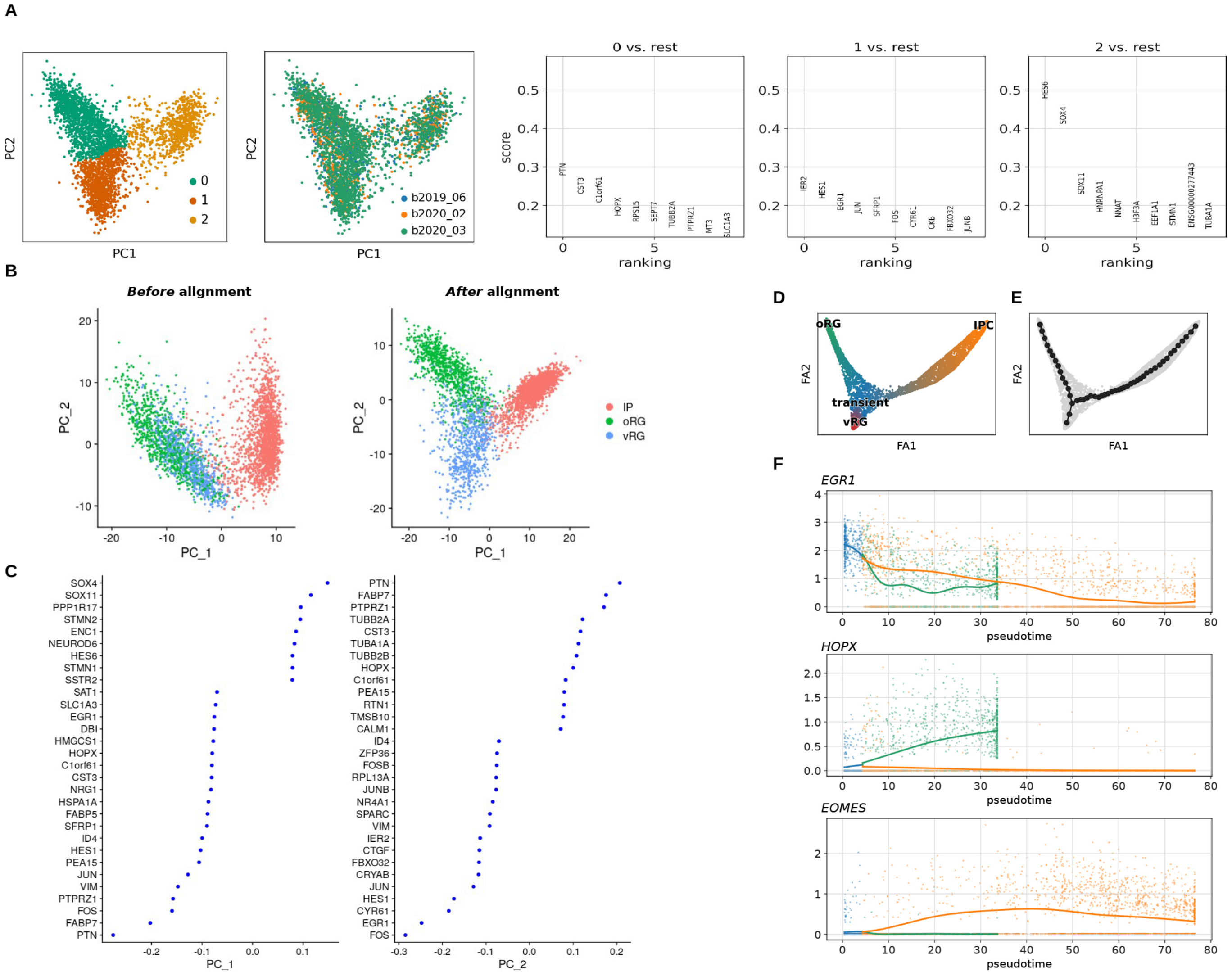

Fig. S1. Differential expression and complementary analysis on an inde-pendent dataset. A) A coarse clustering (leiden algorithm; resolution 0.1) was used for differential gene expression analysis (logistic regression), which captured known markers for each progenitor subtype (0: oRG; 1:vRG; 2:IPC). Additionally, samples from different batches aggregate after normalization and integration (Butler et al., 2018). B) A comparable dataset from (Polioudakis et al., 2019) was used to cross-validate findings obtained with the reference dataset Trevino et al., 2021. Polioudakis et al., 2019 dataset was processed similarly under Seurat analytical framework and projected into a shared low dimensional space, which allowed the discrimination of progenitor subtypes as main axes of variation via principal component analysis. C) Genes that most contribute to the first two principal component analysis in the shared low dimensional space. D) and E) Force-directed graph of neural progenitors from Polioudakis et al., 2019 dataset and projected principal tree on the force-directed graph, respectively. F) Recapitulation of the expected dynamics for three marker genes as pseudotime progresses.

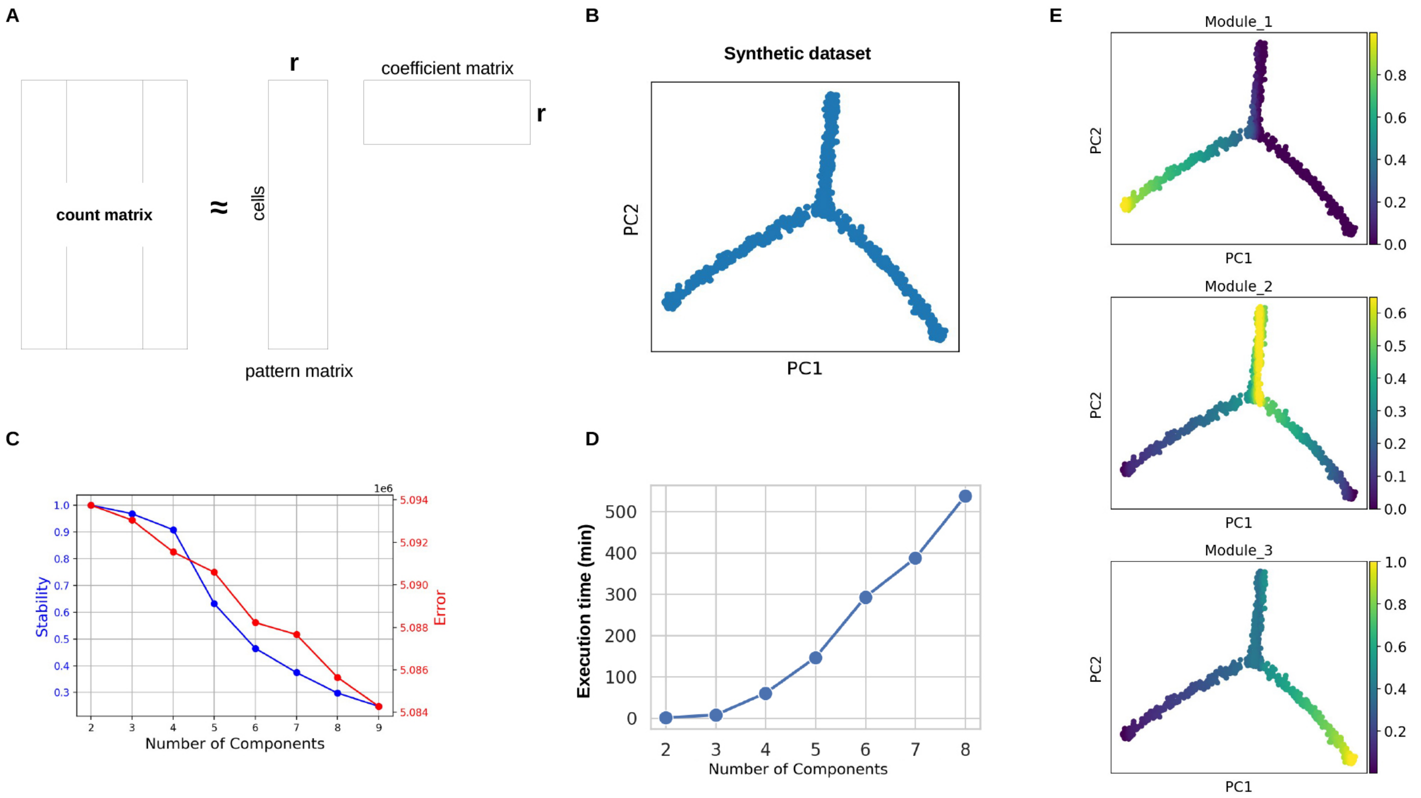

Fig. S2. A) A gene expression matrix, with cells as rows and features as columns, is approximated by two new matrices of lower dimensions. A pattern matrix will capture the cell usage of each of the r inferred gene expression programs, while a coefficient matrix will provide the activation of each feature in each gene expression program. For dynamic trajectories, the expression of each gene can be interpreted as a continuous function on (pseudo)time, and therefore the core algorithm of piNMF (Hautecoeur and Glineur, 2020) aims to learn from the data a set of continuous functions as meaningful dynamic gene expression programs whose number is determined by the factorization rank, r. In order to obtain consensus programs, k-means clustering is used over all replicates (Kotliar et al., 2019). B) A synthetic dataset (Cannoodt et al., 2021) enhances the evaluation of the piNMF implementation, for instance with a simulated bifurfacting trajectory with three meaningful gene expression programs. Analyses on this synthetic dataset can be reproduced with the notebook made available at https://github.com/jjaa-mp/MultiLayered_IndirectNeuro. C) On one hand, a silhouette score provides a stability measure for the r gene expression programs computed after a number of iterations (blue line); on the other hand, the Frobenius norm is used as a cost function to measure the accuracy in the reconstruction of the original gene expression matrix. In this simulated dataset, increasing number of components above four notably reduces the stability of the results, while the error, as expected, decreases as more components are computed. D) The executation time significantly increases as the total number of components to be computed increases. As an estimate, for a synthetic dataset with 2000 cells and 2000 genes, computing a total of eight different components with 200 iterations per component requires above eight hours, with 64Gb of memory available. E) piNMF is able to learn gene expression programs differentially activated on pseudotime and across branches (scale 0 to 1 denotes activation of each gene expression program in each cell).

Development • Supplementary information

Top 10 unique GO terms pseudotime-informed NMF – Modules 2 & 3

oRG_branch_M2	oRG_branch_M3	IPC_branch_M2	IPC_branch_M3
neurogenesis	nervous system development	cell projection organization	nervous system development
brain development	generation of neurons	plasma membrane-bounded cell proj.	system development
anatomical structure development	neurogenesis	cerebellum; molecular layer - neuropil[≥Low]	multicellular organism development
central nervous system development	neuron differentiation	extracellular region	anatomical structure development
columnar/cuboidal epithelial cell differentiation	multicellular organism development	cerebral cortex; neuropil[High]	neurogenesis
neuroepithelial cell differentiation	system development	structural constituent of cytoskeleton	developmental process
forebrain development	membrane	protein binding	generation of neurons
cell population proliferation	anatomical structure development	plasma membrane bounded cell projection	neuron differentiation
central nervous system neuron differentiation	extracellular region	Dysgenesis of the basal ganglia	multicellular organismal process
positive regulation of cell population proliferation	neuron projection development	cellular component morphogenesis	regulation of cellular process

Top 10 unique GO terms standard NMF – Modules 2 & 3

oRG_branch_M2	oRG_branch_M3	IPC_branch_M2	IPC_branch_M3
chromatin organization	Cell Cycle	regulation of cellular process	DNA metabolic process
Abnormality of the palpebral fissures	DNA metabolic process	regulation of biological process	DNA replication
Abnormal lip morphology	DNA replication	positive regulation of developmental process	Cell Cycle
Abnormality of the philtrum	Cell Cycle, Mitotic	chromatin	Cell Cycle, Mitotic
Abnormal upper lip morphology	chromosome	regulation of cell differentiation	DNA-templated DNA replication
Thick eyebrow	chromosome organization	Factor: sp4	cellular response to DNA damage stimulus
Cryptorchidism	cell cycle	Factor: ZXDL	Retinoblastoma gene in cancer
Abnormal eyebrow morphology	cellular response to DNA damage stimulus	biological regulation	DNA repair
Facial hypertrichosis	DNA-templated DNA replication	Factor: ETF	DNA strand elongation
hsa-miR-21-5p	DNA repair	regulation of developmental process	chromosome

Fig. S3. Comparison of GO terms captured by NMF methods for transiently activated modules. Gene expression modules 2 and 3 captured by piNMF are sequentially activated as pseudotime progresses towards basal progenitor cell clusters. GOterms associated to these modules, for either oRG or IP cell clusters, belong to cardinal biological processes relevant for neural progenitor differentiation (upper table), while stdNMF does not fully resolves transient gene expression programs and GO terms are more generic (bottom table). Enrichment analysis was performed using hypergeometric tests (Kolberg et al., 2023) where significant results were considered if corrected p-value < .05

Development • Supplementary information

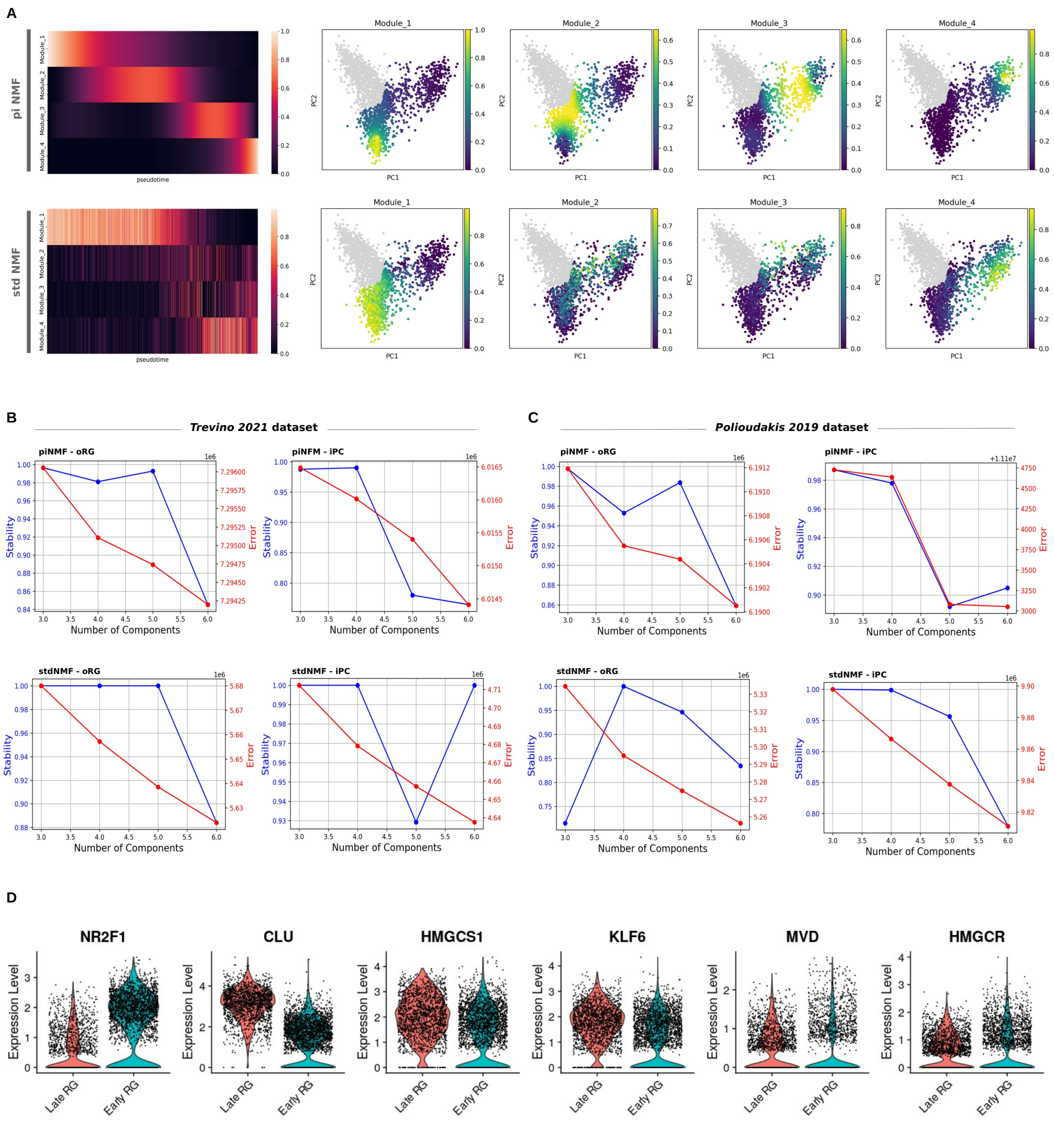

Fig. S4. A) Similarly to the analysis on the oRG branch, piNMF better captures the continuous nature of gene expression programs activated along pseudotime on the IPC branch (see particularly heatmaps on the left), in contrast to stdNMF, specially for transient modules 2 and 3. B) and C) Fac-torization rank selection can be guided by a stability measure (silhouette score) of the resulting components (K-means clustering) over many replicates, and an error metric (Frobenius norm) to evaluate the distance between the original matrix and the NMF approximation. We observed, across branches (vRG to either oRG or IPC), datasets (from Trevino et al., 2021 and Polioudakis et al., 2019) and NMF algorithms (pseudotime-informed and standard NMF) factor-ization rank 4 as a reasonable selection allowing cross-evaluations, according to high stability and decreasing error. As there is not definitive solution for factorization rank selection, a detailed examination of the modules recovered is always required. D) The evaluation of key marker genes (Wilcoxon rank rum test; significant if adj. p-value < 0.01) for cholesterol metabolism highlighted does not reveal a temporal signature among radial glia at neurogenic stages (early vs. late as in (Trevino et al., 2021)); see comparison to markers *NR2F1* and *CLU*.

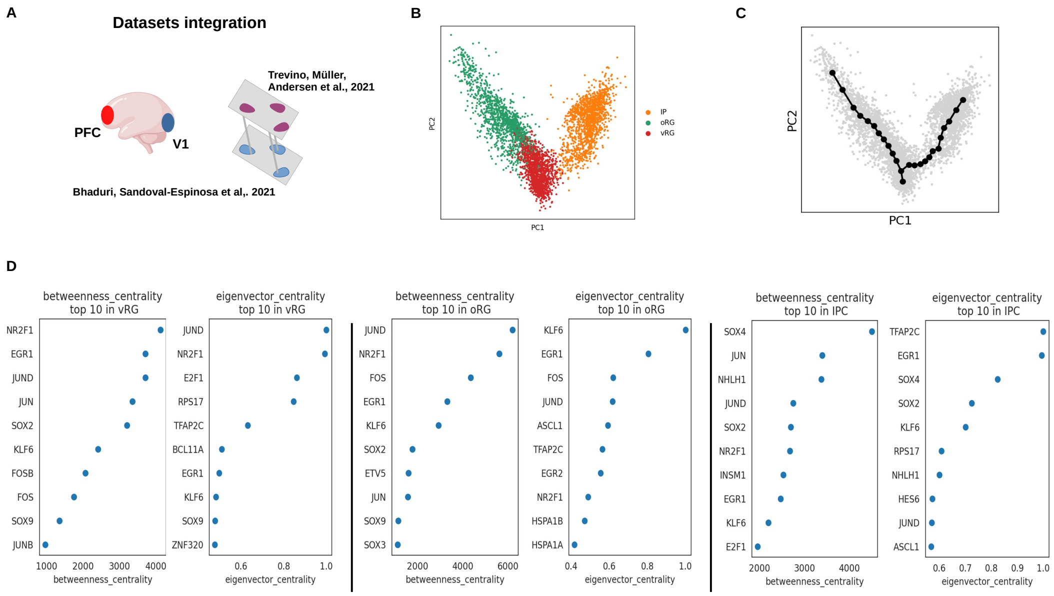

Fig. S5. Non-negative matrix factorization on an integrated dataset. A) Data from reference dataset Trevino et al., 2021 was integrated with spatiotemporally matched PFC and V1 samples from Bhaduri et al., 2021. B) and C) Apical to basal trajectory is capture on the first two dimensions of a principal component analysis as well as the bifurcation among branches on the integrated dataset. D) Gene regulatory network analysis on the integrated dataset recapitulates the prominence of *KLF6* on oRG (top gene on eigenvector centrality scores) in contrast to vRG and IPC. Despite its low expression on IPC and in contrast to results on each independent dataset, *KLF6* does appear in IPC top 10 transcription factors in the integrated dataset.

Development • Supplementary information

A

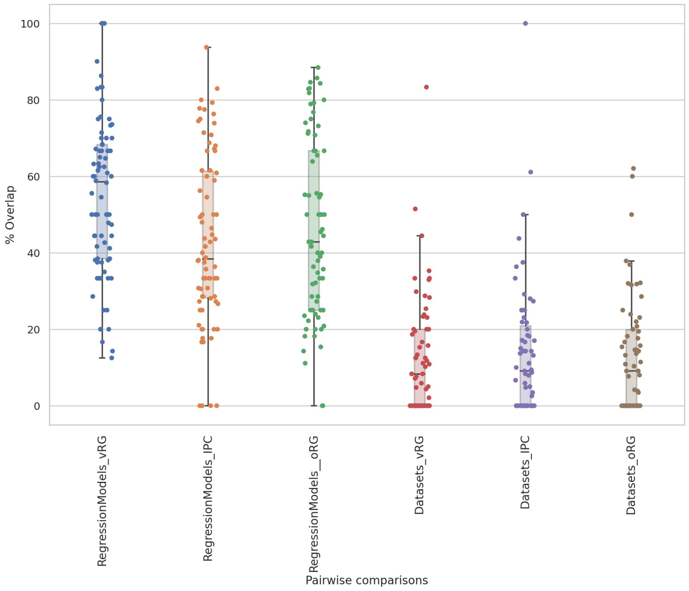

B

Degree distribution (log scale)

Linear regression R^2 values

Cluster	Bagging ridge	Bayesian ridge
**vRG**	0.83	0.79
**oRG**	0.79	0.75
**IPC**	0.86	0.77

Fig. S6. Evaluation of gene regulatory networks across algorithms and datasets. A) Significant overlaps (hypergeometric test; ST5) but substantial variability are detected in the TF-target gene pairs recovered by two machine learning-based Regression Models, bagging ridge and bayesian ridge algorithms from the *CellOracle* software (Kamimoto et al., 2023), when applied to the reference dataset Trevino et al., 2021 (between 43% to 55% depending on the cell cluster). More pronounced differences (overlaps between 12% to 14%) are observed when contrasting GRN Datasets: TF-target gene pairs obtained with *CellOracle* software compared to the regulatory networks (regulons) reported in Polioudakis et al., 2019, a comparable dataset based on *SCENIC* as GRN software (Aibar et al., 2017). B) Among *CellOracle* regression models, the bagging ridge model reports higher linear regression-based R^2 values for the degree distribution of the networks (log scale), and it was our choice for GRN analysis.

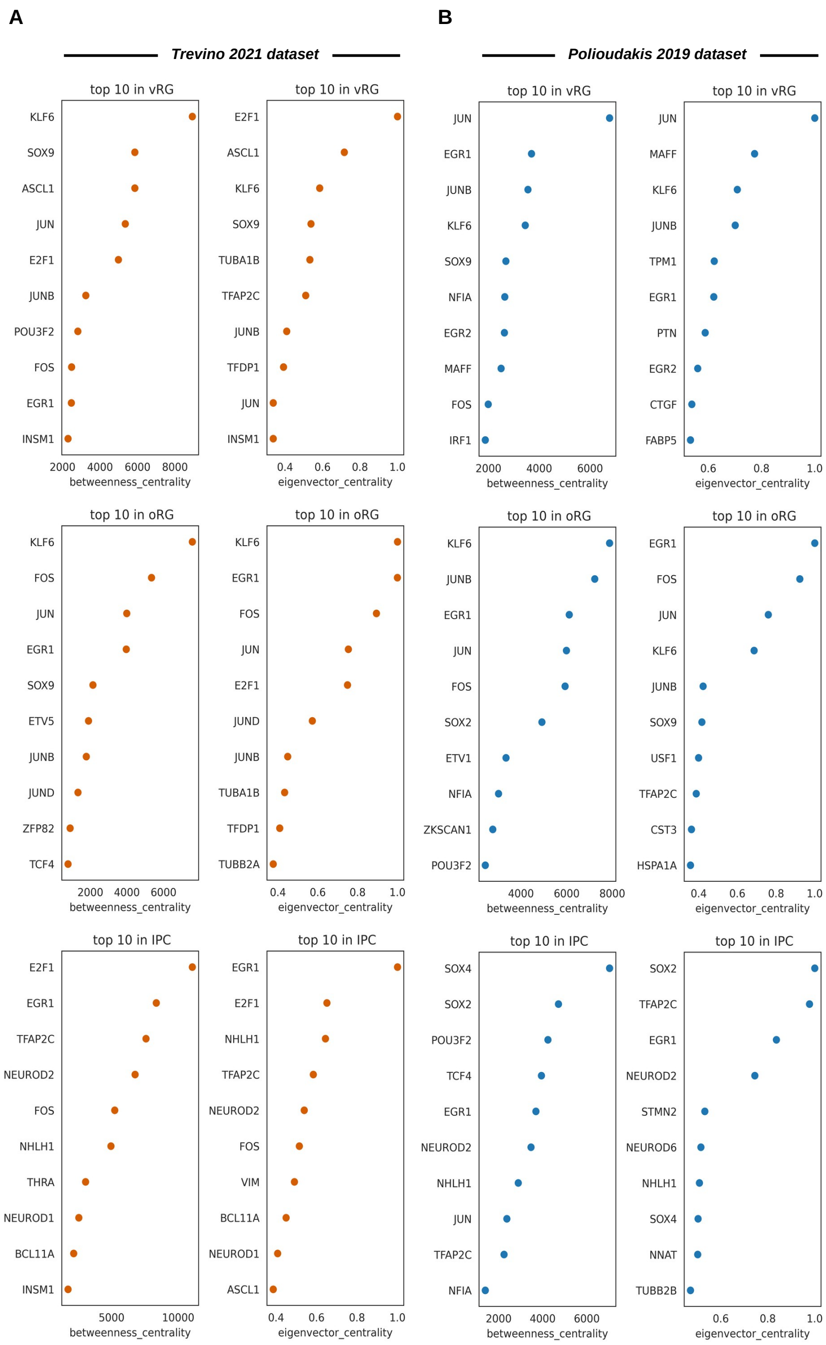

Fig. S7. Networks measures (eigenvector centrality and betweenness central-ity) for two independent datasets. Networks measures (eigenvector centrality and betweenness centrality) for two independent datasets: A) Dataset from Trevino et al., 2021 and B) Dataset from Polioudakis et al., 2019. Genes iden-tified as top 10 in both datasets include *KLF6*, *EGR1*, *JUN*, or *FOS* for radial glial clusters and *NHLH1*, *TFAP2C* or *NEUROD2* in intermediate progenitor clusters.

Development • Supplementary information

References

Aibar, Sara et al. (2017). “SCENIC: single-cell regulatory network inference and clustering”. In: *Nature Methods* 14.11, pp. 1083–1086. doi: 10.1038/nmeth.4463.

Bhaduri, Aparna et al. (2021). “An atlas of cortical arealization identifies dynamic molecular signatures”. In: *Nature* 598.7879, pp. 200–204. doi: 10.1038/s41586-021-03910-8.

Butler, Andrew et al. (2018). “Integrating single-cell transcriptomic data across different conditions, technologies, and species”. In: *Nature Biotechnology* 36.5, pp. 411–420. doi: 10.1038/nbt.4096.

Cannoodt, Robrecht et al. (2021). “Spearheading future omics analyses using dyngen, a multi-modal simulator of single cells”. en. In: *Nature Communications* 12.1. Publisher: Nature Publishing Group, p. 3942. issn: 2041-1723. doi: 10.1038/s41467-021-24152-2. url: https://www.nature.com/articles/s41467-021-24152-2 (visited on 2024).

Hautecoeur, Cécile and François Glineur (2020). “Nonnegative Matrix Factorization over Continuous Signals using Parametrizable Functions”. In: *Neurocomputing* 416, pp. 256–265. doi: 10.1016/j.neucom.2019.11.109.

Kamimoto, Kenji et al. (2023). “Dissecting cell identity via network inference and in silico gene perturbation”. In: *Nature* 614.7949, pp. 742–751. doi: 10.1038/s41586-022-05688-9.

Kolberg, Liis et al. (2023). “g:Profiler—interoperable web service for functional enrichment analysis and gene identifier mapping (2023 update)”. In: *Nucleic Acids Research* 51.W1, W207–W212. issn: 0305-1048. doi:10.1093/nar/gkad347. url: https://doi.org/10.1093/nar/gkad347 (visited on 2024).

Kotliar, Dylan et al. (2019). “Identifying gene expression programs of cell-type identity and cellular activity with single-cell RNA-Seq”. In: *eLife* 8. Ed. by Alfonso Valencia et al., e43803. doi: 10.7554/eLife.43803.

Polioudakis, Damon et al. (2019). “A Single-Cell Transcriptomic Atlas of Human Neocortical Development during Mid-gestation”. In: *Neuron* 103.5, 785–801.e8. doi: 10.1016/j.neuron.2019.06.011.

Trevino, Alexandro E. et al. (2021). “Chromatin and gene-regulatory dynamics of the developing human cerebral cortex at single-cell resolution”. In: *Cell* 184.19, 5053–5069.e23. doi: 10.1016/j.cell.2021.07.039.

Development • Supplementary information

Table S1. Gene ontology enrichment results for NMF gene expression modules - reference dataset.

Available for download at
https://journals.biologists.com/dev/article-lookup/doi/10.1242/dev.202390#supplementary-data

Table S2. Gene ontology enrichment results for NMF gene expression modules – testing dataset and integration.

Available for download at
https://journals.biologists.com/dev/article-lookup/doi/10.1242/dev.202390#supplementary-data

Table S3. Gene ontology enrichment results for cell type-specific KLF6 regulatory networks.

Available for download at
https://journals.biologists.com/dev/article-lookup/doi/10.1242/dev.202390#supplementary-data

Table S4. Gene ontology enrichment results for KLF6 targets across piNMF gene expression modules.

Available for download at
https://journals.biologists.com/dev/article-lookup/doi/10.1242/dev.202390#supplementary-data

Table S5. Comparison gene regulatory networks across datasets and cell types.

Available for download at
https://journals.biologists.com/dev/article-lookup/doi/10.1242/dev.202390#supplementary-data

Table S6. Associated genes to regulatory islands, deserts of introgression, and positively selected regions across early and late gene expression modules.

Available for download at
https://journals.biologists.com/dev/article-lookup/doi/10.1242/dev.202390#supplementary-data

Table S7. Gene ontology enrichment results for TFs impacted by *Homo sapiens*-derived variants.

Available for download at
https://journals.biologists.com/dev/article-lookup/doi/10.1242/dev.202390#supplementary-data

Table S8. TF differential binding affinity analysis results.

Available for download at
https://journals.biologists.com/dev/article-lookup/doi/10.1242/dev.202390#supplementary-data
