# Supplementary material for: A multi-layered integrative analysis reveals a cholesterol metabolic program in outer radial glia with implications for human brain evolution
Source: Development. 2024 Aug 27;151(16):dev202390. doi: 10.1242/dev.202390 (PMC11385646; doi:10.1242/dev.202390)
Supplement: Supplementary file 1 [file develop-151-202390_review_history.pdf]

A multilayered integrative analysis reveals a cholesterol metabolic program in outer radial glia with implications for human brain evolution

Juan Moriano, Oliviero Leonardi, Alessandro Vitriolo, Giuseppe Testa and Cedric Boeckx
DOI: 10.1242/dev.202390

Editor: Debra Silver

Review timeline
Original submission: 1 October 2023
Editorial decision: 16 November 2023
First revision received: 29 April 2024
Editorial decision: 4 June 2024
Second revision received: 1 July 2024
Accepted: 18 July 2024

Original submission

First decision letter

MS ID#: DEVELOP/2023/202390

MS TITLE: A multilayered integrative analysis reveals a cholesterol metabolic program in outer radial glia with implications for human brain evolution

AUTHORS: Juan Moriano, Oliviero Leonardi, Alessandro Vitriolo, Giuseppe Testa, and Cedric Boeckx

I have now received all the referees' reports on the above manuscript, and have reached a decision. The referees' comments are appended below, or you can access them online: please go to BenchPress and click on the 'Manuscripts with Decisions' queue in the Author Area.

As you will see, the referees express considerable interest in your work, but have some significant criticisms and recommend a substantial revision of your manuscript before we can consider publication. If you are able to revise the manuscript along the lines suggested, I will be happy receive a revised version of the manuscript. Reviewer 1 raises concerns about the potential impact of findings (regarding cholesterol genes in oRGs and evolution) and suggests experiments which may help flesh out your findings. Reviewer 2 raises concerns about bioinformatics analyses and need for some validation (suggesting some possible options but others may be suitable). Reviewer 3 requests several important clarifications.

Your revised paper will be re-reviewed by one or more of the original referees, and acceptance of your manuscript will depend on your addressing satisfactorily the reviewers' major concerns. Please also note that Development will normally permit only one round of major revision. If it would be helpful, you are welcome to contact us to discuss your revision in greater detail. Please send us a point-by-point response indicating your plans for addressing the referees' comments, and we will look over this and provide further guidance.

Please attend to all of the reviewers' comments and ensure that you clearly highlight all changes made in the revised manuscript. Please avoid using 'Tracked changes' in Word files as these are lost in PDF conversion. I should be grateful if you would also provide a point-by-point response detailing

© 2024. Published by The Company of Biologists under the terms of the Creative Commons Attribution License (https://creativecommons.org/licenses/by/4.0/).

how you have dealt with the points raised by the reviewers in the 'Response to Reviewers' box. If you do not agree with any of their criticisms or suggestions please explain clearly why this is so.

Reviewer 1

Advance summary and potential significance to field

The mapping of lineage specific variants into cell-type resolved chromatin accessibility landscapes can help revealing the genetic bases of cellular derived features, and the implicated biological processes. In this manuscript Moriano et al. use this approach focusing on the cellular transitions from ventricular radial glial cells to basal progenitors (intermediate progenitors and basal radial glial cells). The authors start by compiling published expression data matched to ATAC data on these cell populations. Next, they classify genes in modules incorporating their expression association with pseudotime in the vRG-oRG, or vRG-IP axes. The resulting modules highlights the enrichment of cholesterol-associated genes in oRG cells. CellOracle is then used to reconstruct gene regulatory networks highlighting KLF6 as highly central in RGc, and inferring multiple targets associated with cholesterol in the oRG network. Finally, authors leverage a set of genomic regions exhibiting modern human-specific variants against the genes previously classified in pseudotime informed modules and enumerate some overlaps. One TF containing such modern-human derived regions is GLI3, a gene that was modeled in CellOracle. The authors find a GLI3 motif in a KLF6 derived region with a disruptive modern human-specific variant, and other variants disrupting TFBS in a GLI3 associated derived region. These observations could potentially connect modern human derived mutations with cholesterol metabolism among other features.

Comments for the author

I found this manuscript very well written and particularly liked the methodology. In particular, the assignation of genes to pseudotime based modules is beautiful and ease the interpretation of modules, compared to other factorization methods. The authors utilize CellOracle effectively to reconstruct cell-type-specific gene regulatory networks. Overall, the methodology is robust and up to date.

Despite all these strengths, I do have some concerns regarding the potential impact of the results, specifically concerning the enhancement of cholesterol-associated gene expression in oRGc and its putative regulation by KLF6.

1) One main finding of this study is the enhance of expression of cholesterol-associated genes in oRGc, and its putative concerted regulation by KLF6.
a. One of the primary findings in this study is the observed increase in expression of cholesterol-associated genes in oRGc. It would be valuable to ascertain whether this upregulation is specific to oRGc or if it correlates with radial glial (RG) maturation in general. It might be useful to investigate whether these genes discriminate between oRGc and vRGc or early RG and later RG. This possibility could be explored using the Trevino et al dataset, which contains samples from multiple donors spanning PCW16 to 24.
b. The relationship between KLF6 and cholesterol genes appears somewhat tenuous based on the available evidence. KLF6 also displays high centrality in vRG, which could indicate its promiscuous nature. If cholesterol genes are upregulated in oRG compared to vRG, it may not be surprising that a promiscuous gene like KLF6 targets them more in oRG. Further investigation is needed to provide a more robust grounding for this proposed relationship.
2) The second main finding is the putative implication of cholesterol genes with human brain evolution.
a. While the authors compile a list of marker genes associated with regulatory regions containing modern-human derived mutations, the connections with KLF6 and cholesterol genes appear somewhat weak and sporadic, particularly in the absence of functional validation.
b. The relationship between KLF6 and GLI3 also appears somewhat speculative. It would be beneficial to investigate whether GLI3 shows differential expression in comparative genomics studies that model early brain development, such as the study conducted by Kanton et al.
c. In general, the section of the paleogenomic interrogation is difficult to follow and lacks clarity regarding the methodology and the results of the intersections. It is unclear whether the

© 2024. Published by The Company of Biologists under the terms of the Creative Commons Attribution License (https://creativecommons.org/licenses/by/4.0/).

specific observations emerge from a systematic exercise or if they are selected to discuss possibly spurious connections between KLF6 and cholesterol genes. Providing a more systematic presentation of the results would help the reader understand better the analysis.

Reviewer 2

Advance summary and potential significance to field

Moriano and co-authors identified an outer radial glia (oRG)-enriched gene expression program and associated transcription factors. Using paleogenomic analysis, they show that this gene program is predicted to be regulated by chromatin regions that were subjected to recent evolutionary changes. In addition the authors showed that cholesterol metabolism is enriched in the oRG gene program.
The findings of the paper, especially implicating cholesterol metabolism in oRG cells, are of potential importance to the field.

Comments for the author

Moriano and co-authors identified an outer radial glia (oRG)-enriched gene expression program and associated transcription factors. Using paleogenomic analysis, they show that this gene program is predicted to be regulated by chromatin regions that were subjected to recent evolutionary changes. In addition, the authors showed that cholesterol metabolism is enriched in the oRG gene program.

The findings of the paper are of potential importance to the field. However, I have some concerns about the way the bioinformatics analysis was conducted. Also, there is no functional validation or functional experiments to demonstrate the effect of identified regulatory regions, transcription factors or effector genes on radial glia morphology or behavior. Below are my specific comments:

1) Insufficient published scRNA-seq and scATAC-seq datasets used for the analysis. There are many published single-cell datasets on the second trimester human cortex: Nowakowski et al Science 2017, Ziffra et al Nature 2021, Bhaduri et al Nature 2021, Herring et al Cell 2022 etc. I think the authors need to integrate datasets from all published second trimester datasets, which will improve the robustness of their analyses.
2) What is the reason the authors used their own approach to identify pseudotime-associated genes? There are several published and well-accepted approaches, such as RNA velocity and Monocle 3. The authors should apply them to their data and show that they can observe the same major findings.
3) It is not completely clear to me how the paleogenomic analysis was performed. How were the regulatory regions defined? Are these known enhancer regions? Which database was used?
4) Lack of statistical measurements. When a certain gene program, such as the oRG one, is claimed to be enriched for recently evolved regulatory interactions, a p value for this enrichment is needed. Same for pathway analysis: is enrichment for cholesterol metabolism significant?
5) Lack of functional validation. I fully appreciate the difficulty of doing functional experiments in human oRG cells. But there are still ways to do it even without access to human tissue. Sliced cerebral organoids have been shown to produce oRG-like cells; knocking out some cholesterol metabolism genes in these or targeting novel transcription factors would be a very important addition to demonstrate the analysis authors performed actually identified functionally relevant targets.

Reviewer 3

Advance summary and potential significance to field

The manuscript from Moriano et al. presents an interesting computational approach to identify gene regulatory networks involved in the differentiation of human neural progenitors during brain

© 2024. Published by The Company of Biologists under the terms of the Creative Commons Attribution License (https://creativecommons.org/licenses/by/4.0/).

development. We appreciate the importance of improving statistical frameworks to analyze and extract interesting patterns from single-cell RNA-seq data sets, and this appears to be the main advance described within this manuscript. To support the importance of this methodological advance the authors present some promising hypotheses, including the involvement of cholesterol metabolism in outer radial glia differentiation, and the importance of KLF6.

Comments for the author

However, we did have some concerns with the manuscript. Broadly, the piNMF method proposed in the manuscript is interesting, but not described or evaluated in sufficient detail. The authors then put forward correlative and speculative analyses. These analyses provide interesting hypotheses, but we felt that some of the arguments would be more convincing if the methods had been better described or the hypotheses were experimentally tested. We believe this manuscript will be of great interest to the readers of Development if our concerns can be largely addressed in a revised manuscript.

Major concerns:
1) The manuscript would benefit from the piNMF method being described in more detail either through text or a figure.
2) The manuscript would also benefit from the piNMF approach being more thoroughly evaluated/described so that readers can understand its strengths and limitations by running on simulated data (or with another approach).
3) We did not notice a software implementation of the method that would allow readers to efficiently reproduce the results in the paper and also use the method on different data sets.
4) In figure 2A which shows how either the stdNMF or piNMF is able to subset gene expression programs into 1 of 4 modules, there is a scale from dark blue to light green. It is not mentioned in the text, on the figure, or in the legend what these colors/numbers mean. We feel that without the scale explained, the reader lacks a deep understanding of what this figure is showing.
5) While we liked the concept of regulatory islands. There are 4836 regulatory islands near 4797 genes, which is about 1/4 or 1/5 of the genes in the genome.
For this reason it was difficult to understand how surprising it was for genes near regulatory islands to be part of various gene sets described in the paper and enrichment may be a better statistic to report. For example, Figure 4B could be strengthened by calculating enrichments of regulatory islands in oRG and IPC gene expression modules over random similarly sized genomic regions instead of reporting percentage overlap. A similar enrichment analysis could be done to see if regulatory islands are enriched for overlaps with regions of positive selection in sapiens as was mentioned.
6) In the section titled “Differential transcription factor binding analysis exhibits signals of positive selection in GLI3 regulatory islands” there is only a quick mention of regulatory islands associated with GLI3 overlap with regions of positive selection identified in a different paper. We feel that these two overlaps need to be described in more detail so that the reader can understand the genetic signals of selection in these areas..
7) We find that Figure 4 is broadly lacking detail, especially in panel C. It is not intuitive what this panel is showing. Based on the manuscript text it seems that certain transcription factor binding sites in regulatory islands are being changed. While the motifs are shown in the figure, it is hard to know how those mutations are predicted to affect binding. A zoomed in region of KLF6 and its associated regulatory island may be more informative of the locus instead of showing a chromosomal scale diagram.

Minor concerns:
1) Twice the authors describe KLF6 as being “unnoticed”. We feel that this may not be the right choice of word, since KLF6 has been studied in other contexts. We think that the authors may want to change to a phrasing that emphasize that KLF6 has been understudied in the context of oRG differentiation
2) Twice (in the abstract and introduction) the authors use the term “(semi)discrete” to describe gene expression programs. It would help readers to provide a definition of this term.
3) In the results detailing regulatory islands, it was occasionally unclear/confusing whether a regulatory island was regulating the gene that was being discussed in cis (e.g. as an enhancer or promoter) or that the mentioned gene was a transcription factor that binds a motif in the

© 2024. Published by The Company of Biologists under the terms of the Creative Commons Attribution License (https://creativecommons.org/licenses/by/4.0/).

regulatory island (regulating a different gene through trans-activation). This is also an issue with figure 4C.
4) Small details in the paleogenomic methods section were stated but not justified.
Why was 3kp picked as the window size for regulatory islands?
Why was the macaque genome used as the ancestral allele if the ancestral state could not be inferred from the multiple alignment? (As opposed to using a more closely related great ape genome or simply throwing out those ambiguous SNPs)

First revision

Author response to reviewers' comments

Responses to editor and reviewers comments
A multi-layered integrative analysis reveals a cholesterol metabolic program in outer radial glia with implications for human brain evolution

Submission ID: DEVELOP/2023/202390

General comments

Reply: We thank the editor and reviewers for their guidance and comments, which helped us bring the manuscript into its current shape. We feel our work has gained in content and clarity of presentation as a result.

We want to apologize for the delay in resubmitting the manuscript. Shortly after receiving the reviews, the first author of the manuscript defended his PhD thesis, and then moved from Europe to the US to start his postdoc position. This was one factor required us to necessitate a longer period to coordinate our reply among all the authors and address all the comments properly.

In the following we address the reviewers' comments and concerns point by point. Our response appears in blue.

Response to the reviewers

Reviewer 1

Reviewer Point P 1.1 — One main finding of this study is the enhance of expression of cholesterol-associated genes in oRGc, and its putative concerted regulation by KLF6.

One of the primary findings in this study is the observed increase in expression of cholesterol- associated genes in oRGc. It would be valuable to ascertain whether this upregulation is specific to oRGc or if it correlates with radial glial (RG) maturation in general. It might be useful to investigate whether these genes discriminate between oRGc and vRGc or early RG and later RG. This possibility could be explored using the Trevino et al dataset, which contains samples from multiple donors spanning PCW16 to 24.

Reply:

Our analysis supports an upregulation of these genes in outer radial glia, while their expression is not exclusive to this cell type. In fact, we already reported in the manuscript the expression of cholesterol genes in ventricular radial glia (Figure 3d). In agreement with the reviewer, we considered of value to further dissect the specificity of cholesterol expression programs in early and late radial glia. To do so, we performed differential expression analysis using the Trevino et al. dataset to compare radial glia across neurogenic stages (from PCW16 to PCW21), and evaluated the expression of key marker genes. Overall, we did not detect significant differences among early versus late radial glia, findings that we now report in Supplementary Figure 4.

Reviewer Point P 1.2 — The relationship between KLF6 and cholesterol genes appears some- what tenuous based on the available evidence. KLF6 also displays high centrality in vRG, which could indicate its

© 2024. Published by The Company of Biologists under the terms of the Creative Commons Attribution License (https://creativecommons.org/licenses/by/4.0/).

promiscuous nature. If cholesterol genes are upregulated in oRG compared to vRG, it may not be surprising that a promiscuous gene like KLF6 targets them more in oRG. Fur- ther investigation is needed to provide a more robust grounding for this proposed relationship.

Reply: This is a very interesting point, and we thank the reviewer for making us think about this more carefully. We believe that there are several reasons why the connection of *KLF6* with cholesterol genes is robust. Both radial glia types express *KLF6*, but with considerably higher eigenvector centrality in outer radial glia. A possible wide and non-selective ("promiscuous") action of KLF6 is hard to reconcile with the following observations: (i) there is a substantial remodeling of KLF6 targets along the radial glia branch (only 22% of targets in oRG are present in vRG), and (ii) such remodeling is accompanied by notable differences in the biological processes over-represented in vRG vs oRG KLF6 regulatory networks. Finally, the specific action of KLF6 in outer radial glia has been reported independently: in Supplementary Material of Polioudakis et al., 2019, KLF6 regulatory network is selectively enriched in oRG and not in other progenitor cells (also in endothelial cells). We of course agree that *KLF6* is not the *only* factor implicated in cholesterol metabolism in radial glia, and we also agree, as the reviewer suggests in the next comment, that ultimately functional validation will be needed.

Reviewer Point P 1.3 — The second main finding is the putative implication of cholesterol genes with human brain evolution.

While the authors compile a list of marker genes associated with regulatory regions contain- ing modern-human derived mutations, the connections with KLF6 and cholesterol genes appear somewhat weak and sporadic, particularly in the absence of functional validation.

The relationship between KLF6 and GLI3 also appears somewhat speculative. It would be beneficial to investigate whether GLI3 shows differential expression in comparative genomics studies that model early brain development, such as the study conducted by Kanton et al.

In general, the section of the paleogenomic interrogation is difficult to follow and lacks clarity regarding the methodology and the results of the intersections. It is unclear whether the specific observations emerge from a systematic exercise or if they are selected to discuss possibly spurious connections between KLF6 and cholesterol genes. Providing a more systematic presentation of the results would help the reader understand better the analysis.

Reply: We have now improved and edited significantly the section titled 'Paleogenomic in- terrogation of regulatory regions active during human corticogenesis'. After the introductory note on regulatory islands discovered from the analysis of the atlas of open chromatin regions, the next two paragraphs present a systematic dissection of regulatory islands. First, a statisti- cal evaluation to answer the question: are regulatory regions associated to genes expressed in IP or oRG lineages over-represented in regulatory islands? And a second statistical evaluation to answer: are regulatory islands found more often than by chance in two sets of genomic re- gions of relevance for the evolution of Homo sapiens (i.e. deserts of introgression and positive selection)? Next, the Transcription Factor Differential Binding Analysis results are presented: (i) overview of increased/decreased TF binding affinities, (ii) Gene Ontology functional evaluation of transcription factors impacted by derived variants and of transcription factors ranked by in- creased affinity; and, finally, (iii) KLF6 regulatory island and GLI3 motif analysis findings. Lastly, we are thankful to the reviewer for pointing to us the study of Kanton et al., in relation to our *GLI3* findings. It is relevant, in the context of our findings, that *GLI3* is reported in that study as being putatively regulated by a differentially accessible region (yet different to our regulatory islands) in humans when compared to chimpanzee cerebral organoids. This is now reported in the Discussion (lines 385-6).

Reviewer 2

Reviewer Point P 2.1 — Insufficient published scRNA-seq and scATAC-seq datasets used for the analysis. There are many published single-cell datasets on the second trimester human cortex: Nowakowski et al Science 2017, Ziffra et al Nature 2021, Bhaduri et al Nature 2021, Herring et al Cell 2022 etc. I think the authors need to integrate datasets from all published second trimester datasets, which will improve the robustness of their analyses.

© 2024. Published by The Company of Biologists under the terms of the Creative Commons Attribution License (https://creativecommons.org/licenses/by/4.0/).

Reply:

We value this suggestion and agree that integration of datasets can indeed strengthen our results. We also think that providing a pipeline for dataset integration can be useful for researchers interested in applying our methodology to their own datasets. We note, however, current limitations in data integration methodologies particularly to retain subtle biological differences across batches.

Following the reviewers' suggestion, we decided to take advantage of the very comprehen- sive atlas of Bahduri, Sandoval-Espinosa et al., 2021, a reference in the field whose data can be easily accessed. We successfully integrated our reference dataset with both PFC and V1 sam- ples at around GW18, a relevant gestational period for the study of indirect neurogenesis and thereby increasing the regional resolution of our analysis. As main results, we could capture the bifurcating trajectory from apical to basal progenitors, and detected dynamic gene expression programs along tree branches. Of note, once again (i) the acquisition of oRG identity is linked to GO terms such as fatty acids, lipids and (ii) KLF6 regulatory networks are linked to choles- terol metabolism, considering significant results if corrected p-value less than 0.05. Overall, this supports our previous results and extend them in scope. We report these new analyses in the relevant sections (lines 185-191; 224-228), with a new figure included as Supplementary Figure 5. In addition, we now provide in our open GitHub repository the code to replicate our analyses and notebooks to facilitate users' implementation of their own dataset integration in the future.

Reviewer Point P 2.2 — What is the reason the authors used their own approach to identify pseudotime-associated genes? There are several published and well-accepted approaches, such as RNA velocity and Monocle 3. The authors should apply them to their data and show that they can observe the same major findings.

Reply: Our aim was to computationally (i) reconstruct a hierarchical structure obtained after pseudotime and bifurcation analyses and (ii) infer the dynamics of gene expression programs on the said hierarchical structure. These are two different but complementary approaches. For the first objective, we used a standard pseudotime approach and lineage-reconstruction algorithm published in Faure et al., 2022. Conveniently, this is implemented in python and therefore the output can be easily used to run our python-based piNMF. Our methodology addresses the sec- ond problem and is framed within an area of research related to matrix factorization approaches.

We believe that the reviewer is right in that we should provide readers with better context to our methodology. In addition to a new Supplementary Figure 2, we address this in the Methods section, lines 470-472, including a citation of a particularly insightful overview: Stein-O'Brien et al., 2018.

Furthermore, following the reviewer's suggestion on comparing results with other approaches, we re-analyzed our data using another pseudotime approach in the CellOracle software. This time we pursued the modelling of cell state trajectories within the CellOracle framework and could correctly infer the transitions from vRG to either oRG or IP, as revealed by the vector field in the PCA-based dimensional space. Building on these results, we considered of relevance to continue exploiting this CellOracle approach to predict the impact of (in silico) knockouts on the said inferred dynamics. We therefore added a new panel in main Figure 3, reporting that the *in silico* ko of *KLF6* causes a shift in cell state trajectories from radial glia towards intermediate progenitors (see also lines 238-241).

Reviewer Point P 2.3 — It is not completely clear to me how the paleogenomic analysis was per- formed. How were the regulatory regions defined? Are these known enhancer regions? Which database was used?

Reply: Our regulatory islands first derive from a consensus set of open chromatin regions active during human neocorticogenesis, replicated across three independent, comprehensive and publicly available datasets from Trevino et al., 2021 Markenscoff-Papadimitriou et al., 2020, and de la Torre-Ubieta et al., 2018, representing high-confidence neocortical cis-regulatory elements. This was then coupled with the catalog of derived mutations published in Kuhlwilm and Boeckx 2019. In order to define uniquely derived regions in the Homo sapiens lineage, we scanned each regu- latory region for Homo sapiens derived variants, and required a standard length of 3kbp around each variant to be depleted of archaic-derived variants. In so doing we required three times a standard enhancer length of 1kb to prevent the inclusion of nearby archaic variants that might have

© 2024. Published by The Company of Biologists under the terms of the Creative Commons Attribution License (https://creativecommons.org/licenses/by/4.0/).

impacted the regulation of gene expression. The consensus set of regulatory regions was then used for gene regulatory network reconstruction and inference of TF-target gene potential interactions.

Reviewer Point P 2.4 — Lack of statistical measurements. When a certain gene program, such as the oRG one, is claimed to be enriched for recently evolved regulatory interactions, a p value for this enrichment is needed. Same for pathway analysis: is enrichment for cholesterol metabolism significant?

Reply: We appreciate the reviewer pointing this out. The relevant information was included in the Supplementary material, but the reviewer is right, it should be indicated more saliently. Each enrichment result highlighted in the text is now accompanied by a statement of significance. Additionally, all p-values can be found in Supplementary Tables. Throughout the manuscript, we only considered significant results if corrected p-values were under .05.

Reviewer Point P 2.5 — Lack of functional validation. I fully appreciate the difficulty of doing functional experiments in human oRG cells. But there are stilll ways to do it even without access to human tissue. Sliced cerebral organoids have been shown to produce oRG-like cells; knocking out some cholesterol metabolism genes in these or targeting novel transcription factors would be a very important addition to demonstrate the analysis authors performed actually identified functionally relevant targets.

Reply: While performing functional validation goes beyond the scope of the present study, we believe that our efforts to provide an in-depth characterization of gene expression programs at play during indirect neurogenesis, and statistical evaluations of species-specific variants with transcription factor differential analaysis, certainly will facilitate experimental interrogations. We fully agree that predictions that emerge from our analyses warrants future functional testing, and state it explicitly in Limitations section. We also point to relevant brain organoid work from one of the author that points to both KLF6 and cholesterol pathway being relevant a a developmental time point that is associated with radial glia production.

Reviewer 3

Reviewer Point P 3.1 — The manuscript would benefit from the piNMF method being described in more detail either through text or a figure.

Reply: We appreciate the encouragement to improve the description of piNMF, and we address this in different ways resulting in changes to text, figures and code (see next comment).

Reviewer Point P 3.2 — The manuscript would also benefit from the piNMF approach being more thoroughly evaluated/described so that readers can understand its strengths and limitations by running on simulated data (or with another approach).

Reply: On the one hand, we added a Supplementary Figure 2 with a visualization of matrix factorization along with a caption to concisely summarize the workings of piNMF. This Supplementary Figure points to the source code and newly generated notebooks that will enable users to go to implement step-by-step a piNMF analysis. On the other hand, a new set of analyses has been added that includes the use of synthetic data to ease the evaluation algorithm parameters and to enhance the interpretability of the results. Specifically, we provide instructions to generate a synthetic bifurcating trajectory and perform gene expression program inference via piNMF, all available through our open GitHub repository referenced in the text. We further exploit this synthetic dataset to facilitate user decisions for exploratory and in-detail analyses using pinNMF; in particular, the trade-off between number of iterations and executation time, as well as reduction of reconstruction errors according to the number of inferred components. The said Supplementary Figure and corresponding caption summarizes these approaches.

© 2024. Published by The Company of Biologists under the terms of the Creative Commons Attribution License (https://creativecommons.org/licenses/by/4.0/).

Reviewer Point P 3.3 — We did not notice a software implementation of the method that would allow readers to efficiently reproduce the results in the paper and also use the method on different data sets.

Reply: Our code can be found at https://github.com/jjaa-mp/MultiLayeredIndirectNeuro/, now complemented with notebooks with step-by-step explanations and synthetic dataset analysis. As stated in these notebooks, a Docker container has now been made available to allow users to easily install core algorithms to reproduce our results and implement a piNMF-based analysis, without the need then of tedious and potentially conflicting local installations.

Reviewer Point P 3.4 — In figure 2A which shows how either the stdNMF or piNMF is able to subset gene expression programs into 1 of 4 modules, there is a scale from dark blue to light green. It is not mentioned in the text, on the figure, or in the legend what these colors/numbers mean. We feel that without the scale explained, the reader lacks a deep understanding of what this figure is showing.

Reply: We agree with the reviewer and now, in the relevant figures, we state that the colorbar represents normalized activation scores (from zero to one) of each gene expression program in each cell.

Reviewer Point P 3.5 — While we liked the concept of regulatory islands. There are 4836 regulatory islands near 4797 genes, which is about 1/4 or 1/5 of the genes in the genome. For this reason it was difficult to understand how surprising it was for genes near regulatory islands to be part of various gene sets described in the paper and enrichment may be a better statistic to report. For example, Figure 4B could be strengthened by calculating enrichments of regulatory islands in oRG and IPC gene expression modules over random similarly sized genomic regions instead of reporting percentage overlap. A similar enrichment analysis could be done to see if regulatory islands are enriched for overlaps with regions of positive selection in sapiens as was mentioned.

Reply: We share the reviewer's point of view and decided to directly address these issues. We would like to point out, as we also caution now in the manuscript, the difficulties of perform- ing these types of statistical evaluations considering that, currently, many algorithms –like the ones we implemented- work best when only a subset of biologically relevant genes (e.g. highly variable genes) are used and, therefore, the tests do not evaluate the full complexity of cell's transcriptomes and epigenomes. We nevertheless consider of relevance the reviewer's sugges- tion and performed permutation tests using random similarly sized genomic regions as control to find statistical associations between genes expressed in each basal progenitor lineage, regula- tory islands, and genomic regions of interest for the recent evolution of our species, as suggested. The results are now included from lines 263 to 281, and description of the methodology in lines 530-532.

Reviewer Point P 3.6 — In the section titled "Differential transcription factor binding analysis exhibits signals of positive selection in GLI3 regulatory islands" there is only a quick mention of regulatory islands associated with GLI3 overlap with regions of positive selection identified in a different paper. We feel that these two overlaps need to be described in more detail so that the reader can understand the genetic signals of selection in these areas.

Reply: We thank reviewer for noticing this part was missing in the manuscript. We now include a description in the Methods section with corresponding references.

Reviewer Point P 3.7 — We find that Figure 4 is broadly lacking detail, especially in panel C. It is not intuitive what this panel is showing. Based on the manuscript text it seems that certain transcription factor binding sites in regulatory islands are being changed. While the motifs are shown in the figure, it is hard to know how those mutations are predicted to affect binding. A zoomed in region of KLF6 and its associated regulatory island may be more informative of the locus instead of showing a chromosomal scale diagram.

Reply: We agree with the reviewer that Figure 4 could be improved. We have substantially upgraded Figure 4 with improved quality of figures, adding relevant information such as the GO

© 2024. Published by The Company of Biologists under the terms of the Creative Commons Attribution License (https://creativecommons.org/licenses/by/4.0/).

enrichment analysis, as well as including a new visualization with a zoom-in for *KLF6* region as suggested by the reviewer.

Reviewer Point P 3.8 — Minor concerns:

1) Twice the authors describe KLF6 as being "unnoticed". We feel that this may not be the right choice of word, since KLF6 has been studied in other contexts. We think that the authors may want to change to a phrasing that emphasize that KLF6 has been understudied in the context of oRG differentiation.

Reply: We removed the statements 'previously unnoticed'. We think our statement in the Discussion section regarding KLF6, 'whose role in human neurogenesis has to date remained largely undescribed', aligns well with the reviewer's suggestion.

Reviewer Point P 3.9 — Twice (in the abstract and introduction) the authors use the term "(semi)discrete" to describe gene expression programs. It would help readers to provide a defini- tion of this term.

Reply: We agree that this was not clear in the manuscript. We use (semi)discrete to emphasize the fact that our partition of gene expression profiles into different components allow genes to be found in more than one component, instead of using a strict exclusion criterion for gene module membership. This is in line with the expectation that genes upregulated early on might be upregulated at later timepoints. The identification of top marker genes using multiple least squares regression sets a less strict criterion for gene assignments to modules. We added the following statement: 'We refer to '(semi)discrete modules' to note that genes might be present in more than one module to the extent the association is statistically significant (higher expression than the rest of genes in cells with activation of the given expression module)' (lines 506-508).

Reviewer Point P 3.10 — In the results detailing regulatory islands, it was occasionally unclear/confusing whether a regulatory island was regulating the gene that was being discussed in cis (e.g. as an enhancer or promoter) or that the mentioned gene was a transcription factor that binds a motif in the regulatory island (regulating a different gene through trans-activation). This is also an issue with figure 4C.

Reply: We have significantly edited the section A paleogenomic interrogation of regulatory regions active during human corticogenesis, also Figure 4, aiming at presenting the results in a more logical and clear manner.

Reviewer Point P 3.11 — Small details in the paleogenomic methods section were stated but not justified. Why was 3kp picked as the window size for regulatory islands? Why was the macaque genome used as the ancestral allele if the ancestral state could not be inferred from the multiple alignment? (As opposed to using a more closely related great ape genome or simply throwing out those ambiguous SNPs)

Reply: We now extend our choices in the Method section. In particular, regarding the window size of regulatory islands, we required three times a standard enhancer length of 1kb to exclude nearby Neanderthal/Denisovan archaic variants that might have also impacted the regulation of the predicted target gene. Regarding the other question (the use of the macaque genome), we followed the criteria chosen and justified in Kuhlwilm and Boeckx 2019. We refer the reviewer to that publication for further discussion.

<u>Second decision letter</u>

MS ID#: DEVELOP/2023/202390

MS TITLE: A multilayered integrative analysis reveals a cholesterol metabolic program in outer radial glia with implications for human brain evolution

© 2024. Published by The Company of Biologists under the terms of the Creative Commons Attribution License (https://creativecommons.org/licenses/by/4.0/).

AUTHORS: Juan Moriano, Oliviero Leonardi, Alessandro Vitriolo, Giuseppe Testa, and Cedric Boeckx

I have now received all the referees reports on the above manuscript, and have reached a decision. The referees' comments are appended below, or you can access them online: please go to BenchPress and click on the 'Manuscripts with Decisions' queue in the Author Area.

The overall evaluation is positive and we would like to publish a revised manuscript in Development, provided that the referees' comments can be satisfactorily addressed. Please attend to all of the reviewers' comments in your revised manuscript and detail them in your point-by-point response. If you do not agree with any of their criticisms or suggestions explain clearly why this is so.

Specifically, please address remaining concerns of reviewer 2 regarding figures and reviewer 1 by including a reference and discussion of the new Figure S4 in the results. In addition, please include in the discussion the specificity of relationship between cholesterol genes in oRGs and KLF6 (paraphrasing or including verbiage from your response about this). This reviewer has a lingering concern about the evolutionary analysis. Should you be able to include additional transcription factor analysis this would be valuable however I do not view it as essential. I request that in addition to including the point by point response, please also include a tracked changes word document with this submission.

Reviewer 1

Advance summary and potential significance to field

The authors found an enrichment in cholesterol genes among targets of KLF6, a transcription factor they found to display high network centrality within RGC particularly in oRGC.

Comments for the author

The authors have addressed my point regarding the oRGC vs. late RGC KLF5 expression by adding a new supplementary figure. This figure shows no significant differences between early and later RGCs from Trevino et al. However, there is no P-value or fold change mentioned for these comparisons. Visually, KLF6 seems to be more expressed in late RG than in early RG. Moreover, this analysis is not mentioned in the text.

I raised concerns about the specificity of the relationship between cholesterol genes in oRGCs and KLF6, given KLF6's high expression in RG. The authors argue that target differences between networks in vRG and oRG indicate KLF6 promiscuity does not explain the differences between both types. However, it seems that comparisons with the Polioudakis et al. 2019 dataset show similar enrichment in both types of RGCs.

My third concern related to the clarity of the paleogenomics analysis of human-specific variants. Although the authors have systematized the analysis, I still find this section somewhat disconnected from the KLF6-cholesterol story. It appears to be an independent analysis that is later linked to KLF6 through sporadic findings in a few genes, some of which are related to cholesterol. A more focused analysis on KLF6 targets with disrupted KLF6 motifs and checking for an enrichment in cholesterol genes among these divergent targets would be more cohesive.

Reviewer 2

Advance summary and potential significance to field

The authors address the comments from my previous review, and I believe the paper is improved.

Comments for the author

© 2024. Published by The Company of Biologists under the terms of the Creative Commons Attribution License (https://creativecommons.org/licenses/by/4.0/).

Thank you for addressing my comments and adding data from another dataset to validate your findings. Two minor comments: 1) Please use the same colors for vRG, oRG and IP in Figure 2—figure supplement 4 and other figures, 2) Make sure to state how integration was done and add Methods section to explain the methodology (right now, it is not clear whether it was done using SCTransform and IntegrateData or some other approach).

Reviewer 3

Advance summary and potential significance to field

The manuscript from Moriano et al. presents an interesting computational approach to identify gene regulatory networks involved in the differentiation of human neural progenitors during brain development. We appreciate the importance of improving statistical frameworks to analyze and extract interesting patterns from single-cell RNA-seq data sets, and this appears to be the main advance described within this manuscript. To support the importance of this methodological advance the authors present some promising hypotheses, including the involvement of cholesterol metabolism in outer radial glia differentiation, and the importance of KLF6.

Comments for the author

The authors' responses to my comments are acceptable.

Second revision

Author response to reviewers' comments

Responses to editor and reviewers comments

A multi-layered integrative analysis reveals a cholesterol metabolic program in outer radial glia with implications for human brain evolution

Submission ID: DEVELOP/2023/202390

General comments

Reply: We wish to thank once more the editor and reviewers for their positive evaluation and comments, helping us to further improve the manuscript, and introduce further clarity. Here below we address the reviewers' comments, following the editor's guidance.

Changes in the manuscript are now highlighted with Microsoft Word Track Changes, as per the instructions we received.

Response to the reviewers

Reviewer 1

Reviewer Point P 1.1 — The authors have addressed my point regarding the oRGC vs. late RGC KLF5 expression by adding a new supplementary figure. This figure shows no significant differences between early and later RGCs from Trevino et al. However, there is no P-value or fold change mentioned for these comparisons. Visually, KLF6 seems to be more expressed in late RG than in early RG. Moreover, this analysis is not mentioned in the text.

I raised concerns about the specificity of the relationship between cholesterol genes in oRGCs and KLF6, given KLF6's high expression in RG. The authors argue that target differences between networks in vRG and oRG indicate KLF6 promiscuity does not explain the differences between both types. However, it seems that

© 2024. Published by The Company of Biologists under the terms of the Creative Commons Attribution License (https://creativecommons.org/licenses/by/4.0/).

comparisons with the Polioudakis et al. 2019 dataset show similar enrichment in both types of RGCs.

My third concern related to the clarity of the paleogenomics analysis of human- specific vari- ants. Although the authors have systematized the analysis, I still find this section somewhat disconnected from the KLF6-cholesterol story. It appears to be an independent analysis that is later linked to KLF6 through sporadic findings in a few genes, some of which are related to choles- terol. A more focused analysis on KLF6 targets with disrupted KLF6 motifs and checking for an enrichment in cholesterol genes among these divergent targets would be more cohesive.

Reply:

We thank the reviewer for raising these points. We have now added a reference in the main text to the differential expression test, Supplementary Figure S4, and a description in the Meth- ods section. The code for the analysis can also be found at our dedicated GitHub repository. Re- garding the second issue, namely the relationship between *KLF6* and cholesterol genes in radial glia, our implementation of different analytical approaches, applied on independent datasets, point to the prominence of *KLF6* in the regulation of cholesterol genes specifically upon the ac- quisition of oRG fate, further supported by complementary evidence we report in the Discussion section. Overall, we agree with the reviewer that the possible differences in *KLF6* functional roles in radial glia subtypes requires further investigation. We make this point further clearer in the Discussion section, as also suggested by the editor. As for the third issue raised, we have tried at all stages to be quite cautious throughout the manuscript to state our findings lead to an hypothesis, to be validated or disproved in the future, and we think that our current analyses provide enough resolution to experimentally address reviewer's concerns, including the pertur- bation of *KLF6* expression in human radial glia, the evaluation of the *KLF6* mutation predicted to impact GLI3 binding, as well as unraveling the transcriptional networks impacted by such possible cross-regulation.

We really appreciate the reviewer´s assistance to make us think further about the manuscript´s conclusions and to significantly improve the clarity of our ideas and analysis.

Reviewer 2

Reviewer Point P 2.1 — Thank you for addressing my comments and adding data from another dataset to validate your findings. Two minor comments: 1) Please use the same colors for vRG, oRG and IP in Figure 2—figure supplement 4 and other figures, 2) Make sure to state how inte- gration was done and add Methods section to explain the methodology (right now, it is not clear whether it was done using SCTransform and IntegrateData or some other approach).

Reply:

We thank the reviewer for pointing out to us the need for these corrections. We corrected the current Supplementary Figure S5 as well as described the Integration procedure, referenc- ing Bhaduri et al., 2021 dataset and main functions used (Single-cell RNA-seq data processing subsection of Methods section).

Reviewer 3

Reviewer Point P 3.1 — The authors' responses to my comments are acceptable.

Reply: We appreciate all comments and insights from reviewer 3.

<u>Third decision letter</u>

MS ID#: DEVELOP/2023/202390

© 2024. Published by The Company of Biologists under the terms of the Creative Commons Attribution License (https://creativecommons.org/licenses/by/4.0/).

MS TITLE: A multilayered integrative analysis reveals a cholesterol metabolic program in outer radial glia with implications for human brain evolution

AUTHORS: Juan Moriano, Oliviero Leonardi, Alessandro Vitriolo, Giuseppe Testa, and Cedric Boeckx
ARTICLE TYPE: Research Article

I am happy to tell you that your manuscript has been accepted for publication in Development, pending our standard publication integrity checks.

© 2024. Published by The Company of Biologists under the terms of the Creative Commons Attribution License (https://creativecommons.org/licenses/by/4.0/).
